# Non-canonical features of the Golgi apparatus in bipolar epithelial neural stem cells

**DOI:** 10.1038/srep21206

**Published:** 2016-02-16

**Authors:** Elena Taverna, Felipe Mora-Bermúdez, Paulina J. Strzyz, Marta Florio, Jaroslav Icha, Christiane Haffner, Caren Norden, Michaela Wilsch-Bräuninger, Wieland B. Huttner

**Affiliations:** 1 Max-Planck Inst. of Mol. Cell Biol. and Genetics, Pfotenhauerstr. 108, 01307 Dresden, Germany

## Abstract

Apical radial glia (aRG), the stem cells in developing neocortex, are unique bipolar epithelial cells, extending an apical process to the ventricle and a basal process to the basal lamina. Here, we report novel features of the Golgi apparatus, a central organelle for cell polarity, in mouse aRGs. The Golgi was confined to the apical process but not associated with apical centrosome(s). In contrast, in aRG-derived, delaminating basal progenitors that lose apical polarity, the Golgi became pericentrosomal. The aRG Golgi underwent evolutionarily conserved, accordion-like compression and extension concomitant with cell cycle-dependent nuclear migration. Importantly, in line with endoplasmic reticulum but not Golgi being present in the aRG basal process, its plasma membrane contained glycans lacking Golgi processing, consistent with direct ER-to-cell surface membrane traffic. Our study reveals hitherto unknown complexity of neural stem cell polarity, differential Golgi contribution to their specific architecture, and fundamental Golgi re-organization upon cell fate change.

The primary neural stem cells from which all other cells of the mammalian central nervous system (CNS) are derived, are canonical epithelial cells^1^. These cells, called neuroepithelial cells, exhibit apical-basal cell polarity and contact a basal lamina with their basal plasma membrane and the lumen of the brain ventricles and spinal cord central canal with their apical plasma membrane. Apical and basolateral plasma membrane domains are separated from each other by a belt of cell junctions at the apical-most end of the basolateral membrane that are crucial for maintaining the cells integrated into the neuroepithelium^2^. During interphase, a primary cilium protrudes from the apical plasma membrane of neuroepithelial cells into the lumen. The membrane association of the ciliary basal body, that is, the mother centriole, is responsible for the interphase centrosome(s) being tethered at the apical plasma membrane^3^.

Early in CNS development, the neuroepithelium consists of a single layer of neuroepithelial cells that exhibits pseudostratification because the nuclei occupy various positions along the apical-basal axis. This reflects a process called interkinetic nuclear migration (INM)^4^,^5^. Following mitosis just beneath the apical plasma membrane, nuclei migrate basally during the G1 phase of the cell cycle such that S-phase takes place near the basal lamina. During G2, nuclei migrate in the opposite direction towards the apically tethered centrosomes and then undergo again apical mitosis. At the early developmental stage, all divisions of neuroepithelial cells are symmetric proliferative, that is, both daughters are neuroepithelial cells.

With the onset of neurogenesis, neuroepithelial cells transform into a highly related, but nonetheless distinct, cell type called apical radial glia (aRG)^6^. As not only neuroepithelial cells, but also aRGs undergo apical mitosis, they are collectively referred to as apical progenitors (APs). The transformation from neuroepithelial cells to aRGs is accompanied by several substantial changes that are most pronounced in the developing neocortex and pertain to the mode of cell division and daughter cell fate, and consequently to the cell biology, INM, and tissue architecture. Specifically, neuroepithelial cells and subsequently aRGs switch to asymmetric self-renewing division, which generates an aRG daughter and a daughter cell with a different fate that delaminates from the apical surface and junctional belt, loses apical cell polarity features, and migrates basally to generate additional cell layers. In the developing neocortex, this basal daughter cell can be a neuron, but in most cases is a secondary type of stem or progenitor cell, collectively referred to as basal progenitors (BPs)^7^,^8^,^9^, which ultimately generate most cortical neurons^10^.

With the generation of BPs and neurons, the developing cortical wall changes from a pseudostratified epithelium to a mixed, pseudostratified-stratified, epithelium, as not all of the newly generated cells are in contact with the basal lamina. The aRG nuclei are now confined to the apical-most zone, called ventricular zone (VZ). BPs form another germinal layer basal to the VZ, the subventricular zone (SVZ). Newborn neurons produced by BPs migrate from the SVZ towards the basal lamina to form the basal-most cell layers, the cortical plate (CP).

Importantly, despite the formation of the SVZ and CP basal to the VZ, the aRGs maintain their contact with the basal lamina through a long thin process that traverses SVZ and CP, called basal process. In addition, aRGs also maintain contact with the ventricle through an apical process and remain integrated in the apical junctional belt. Because of this cytoarchitecture, aRGs represent unique bipolar epithelial cells. Specifically, the cytoplasm bounded by their basolateral plasma membrane, which as such spans the entire cortical wall, actually constitutes two distinct compartments, the apical process that spans all of the VZ, and the basal process that spans all layers basal to the VZ. Of note, aRGs continue to exhibit cell cycle-dependent INM, but given that aRG nuclei reside in the VZ, this nucleokinesis is now confined to the apical process.

These features of aRGs raise several fundamental cell biological questions. First, are there differences in subcellular organization between the apical and basal process of aRGs? If so, do they explain why the apical, but not the basal, process is permissive for INM? Second, what happens to the organelles within the apical process during INM? Third, are there differences, in principle, in the plasma membrane constituents of the apical versus basal process? If so, are there differential delivery routes for plasma membrane constituents to the apical versus basal process? And fourth, what are the changes in subcellular organization, in comparison to aRGs, in their BP daughters as these delaminate from the apical surface and junctional belt, lose apical cell polarity features, and migrate to the SVZ? In the present study, we have addressed these questions, of relevance for both cell biology and stem cell biology, by focussing on the Golgi apparatus, a ubiquitous organelle in all eukaryotes with a central role in the processing and sorting of newly synthesized secretory and membrane constituents. In particular, the Golgi apparatus has key functions in establishing and maintaining epithelial cell polarity^11^,^12^. Of note, in mammals, the Golgi apparatus is known to be associated with the centrosome^13^. This association is not only of a physical, but also functional nature, as both the Golgi apparatus and the centrosome are linked to cell cycle progression, polarized cell migration, and ciliogenesis^14^,^15^,^16^,^17^.

We find that, remarkably, the Golgi apparatus in aRGs is not associated with the centrosome, is confined to the apical process and the cytoplasm apical to the nucleus, alters its extension in concert with the cell cycle-dependent INM, and has only a minor role in the delivery of plasma membrane constituents to the basal process. Upon BP delamination, the Golgi apparatus resumes its canonical pericentrosomal location. These findings point to a crucial role of the Golgi apparatus in maintaining neural stem cell identity and their cytoarchitecture during the development of the mammalian neocortex.

## Results

### The Golgi apparatus of apical radial glia is confined to their apical process

**Figure 1:**
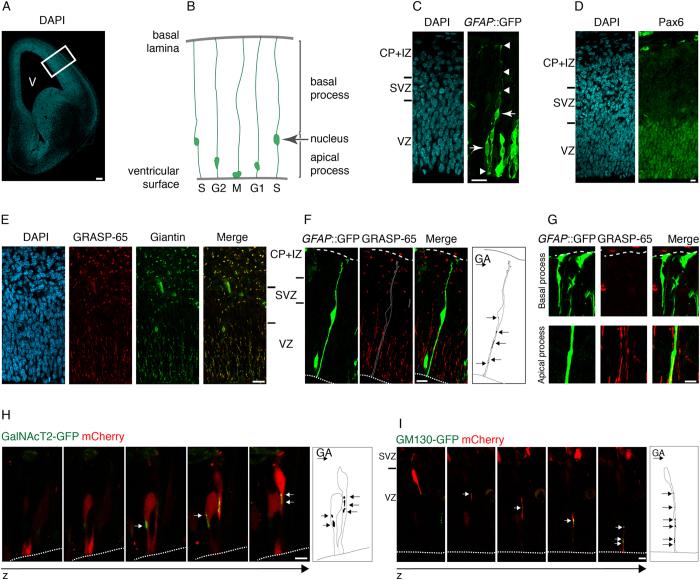
Subcellular localization of the Golgi apparatus in aRG. (**A**) DAPI-stained E13.5 wt mouse telencephalon; single 1.5-μm optical section. Box: region of dorsolateral telencephalon examined in this study; V, ventricle. Scale bar, 100 μm. (**B**) Cartoon illustrating major features of aRG morphology and INM. (**C**) aRG in dorsolateral telencephalon of E13.5 *GFAP*::GFP transgenic mouse embryo, identified by GFP immunofluorescence (green, stack of 30 0.8-μm optical sections) combined with DAPI staining (blue, single 0.8-μm optical section at z-position of the indicated nuclei); arrowheads: apical process contacting ventricle (bottom) and basal process reaching the basal lamina (top). Scale bar, 20 μm. IZ, intermediate zone; in (**C-F**), the CP and IZ are indicated together; in the following figures, these layers are indicated as CP only. (**D**) aRG nuclei in VZ of dorsolateral telencephalon of E13.5 wt mouse embryo immunostained for Pax6 (green) combined with DAPI staining (blue, 0.8-μm optical sections). Scale bar, 10 μm. (**E**) Golgi apparatus in dorsolateral telencephalon of E14.5 wt mouse embryo revealed by GRASP-65 (red) and giantin (green) double immunofluorescence combined with DAPI staining (blue, 0.8-μm optical sections). Scale bar, 20 μm. (**F**) Subcellular localization of Golgi apparatus, revealed by GRASP-65 immunofluorescence (red), in an individual aRG in dorsolateral telencephalon of E13.5 *GFAP*::GFP transgenic mouse embryo, identified by GFP immunofluorescence (green, stack of 13 0.8-μm optical sections). Diagram: localization of all Golgi units (arrows) in the cell as reconstructed from the series of optical sections. Dashed lines, ventricular surface (bottom) and basal lamina (top). Scale bar, 10 μm. (**G**) Bottom row: presence of Golgi apparatus in the apical process of the aRG shown in (**F**). Top row: absence of Golgi apparatus in the basal process of another aRG, analyzed as in (**F**); dashed lines, basal lamina. Scale bars, 2 μm. (**H,I**) Z-stacks of APs in VZ of dorsolateral telencephalon of E14.5 wt mouse embryo co-electroporated *in utero* at E13.5 with plasmids encoding mCherry and either GalNAcT2-GFP (**H**) or GM130-GFP (**I**). Images are consecutive 0.8 μm optical sections. Diagrams: localization of all Golgi units (arrows) in the respective APs. Dashed lines, ventricular surface. Scale bars, 10 μm.

To study the Golgi apparatus in single aRG in the developing mouse neocortex (Fig. 1A), we made use of the transgenic *GFAP::*GFP mouse line, in which cytosolic GFP is expressed under the control of the human GFAP promoter^18^. In *GFAP::*GFP mouse embryos at mid-neurogenesis, GFP is expressed by only a subset of aRG, which allows the identification of single aRG including their apical and basal processes^19^,^20^ (Fig. 1B,C). The nuclei of aRG, which can be identified by staining for the transcription factor Pax6 (Fig. 1D), are known to reside in the VZ and to occupy different positions depending on the phase of the cell cycle (INM, Fig. 1B).

To identify the Golgi apparatus, we performed immunocytochemistry for a well-established Golgi marker, GRASP-65, a Golgi-stacking protein enriched in the cis-to-medial Golgi^21^,^22^. Immunofluorescence of the E13.5 mouse cortical wall showed GRASP-65 immunoreactivity in all cortical zones, with a radial pattern in the VZ (Fig. 1E). The specificity of the GRASP-65 staining was corroborated by (i) double immunofluorescence for giantin, a golgin protein enriched in the medial-to-trans Golgi^23^ that showed a very similar staining pattern to GRASP-65 (Fig. 1E); and (ii) GRASP-65 immuno-gold electron microscopy, which showed immunoreactivity associated with Golgi cisternae (Supplementary Figure S1).

Analysis of GRASP-65 immunoreactivity in single, *GFAP::*GFP–positive aRG revealed the following. First, the Golgi apparatus is excluded from the basal process of aRG (Fig. 1F,G, top). Second, the Golgi apparatus is found in the aRG cell body (that is, the portion of the apical process where the nucleus happens to be (Fig. 1B)) and in the portion of the apical process between the nucleus and the apical surface (Fig. 1F,G, bottom). Third, the Golgi apparatus is not found in the portion of the apical process that is basal to the nucleus (see Fig. 1B). Fourth, the Golgi apparatus appears to consist of multiple units (for quantification, see below) that do not seem to be connected to each other (Fig. 1F,G, bottom). The same observations were made when the Golgi-resident proteins GalNAcT and GM130 were exogenously expressed as GFP-tagged fusion proteins in single aRG by *in utero* electroporation (Fig. 1H,I).

### The Golgi apparatus in the apical process consists of separate stacks, with the cis-trans axis perpendicular to the apical-basal axis

**Figure 2:**
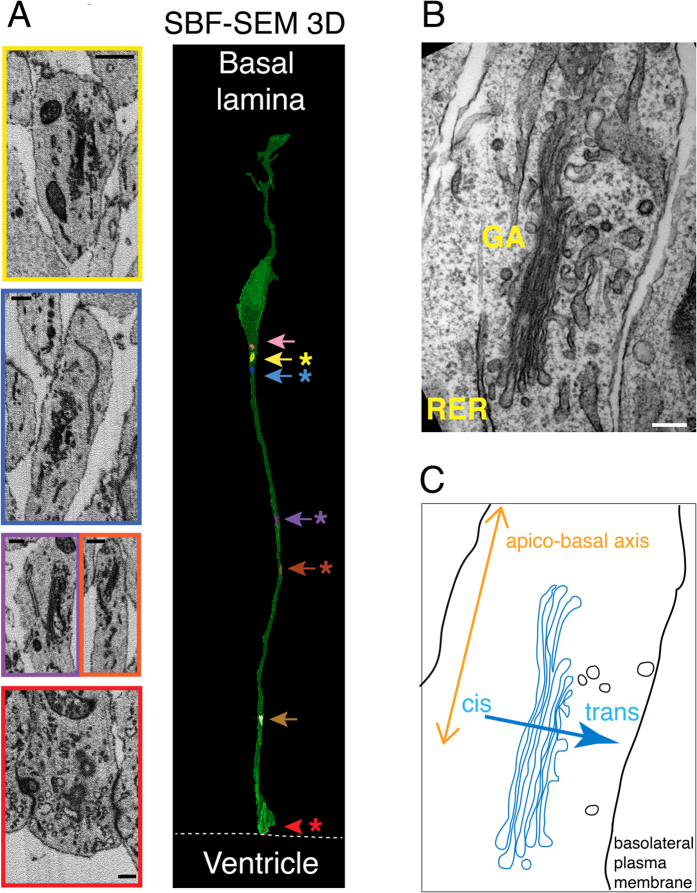
Electron microscopy analyses of the Golgi apparatus in aRG. (**A**) SBF-SEM 3D reconstruction of an individual aRG (right) in E12.5 wt mouse dorsolateral telencephalon. Green, cell volume; arrows, Golgi units; arrowhead, centrosome. Asterisks indicate areas shown as electron micrographs on the left; note the color-coding. Dashed line, ventricular surface. Scale bars, 500 nm. (**B**) Transmission electron micrograph of a Golgi stack in the apical process of an aRG in E12.5 wt mouse dorsolateral telencephalon. Note the orientation of the cis-to-trans polarity axis of the Golgi apparatus (GA) perpendicular to the long axis of the apical process, i.e. the apico-basal axis of the cell. RER, rough endoplasmic reticulum. Scale bar, 100 nm. (**C**) Cartoon illustrating the orientation of the Golgi apparatus shown in (**B)**.

To gain further insight into the organization of the Golgi apparatus within the apical process of aRG, we used serial block face scanning electron microscopy (SBF-SEM), the imaging method of choice for the three-dimensional reconstruction of subcellular structures^24^,^25^. Figure 2A shows a single aRG containing six separate Golgi apparatus stacks in the apical process. All of the Golgi stacks analyzed were (i) distributed between the nucleus and the apical plasma membrane (Fig. 2A), and (ii) oriented with their cis-to-trans axis perpendicular to the apical-basal axis of the cell (106 of 106 stacks; see Fig. 2B,C). In all aRG analyzed and reconstructed, Golgi stacks were observed apically (and rarely also lateral) to the nucleus, and no membrane structures identifiable as Golgi cisternae were observed within the basal process (Fig. 2A), consistent with the lack of GRASP-65 immunoreactivity in the basal process (Fig. 1F,G, top).

### The extension of the Golgi apparatus is linked to interkinetic nuclear migration

**Figure 3:**
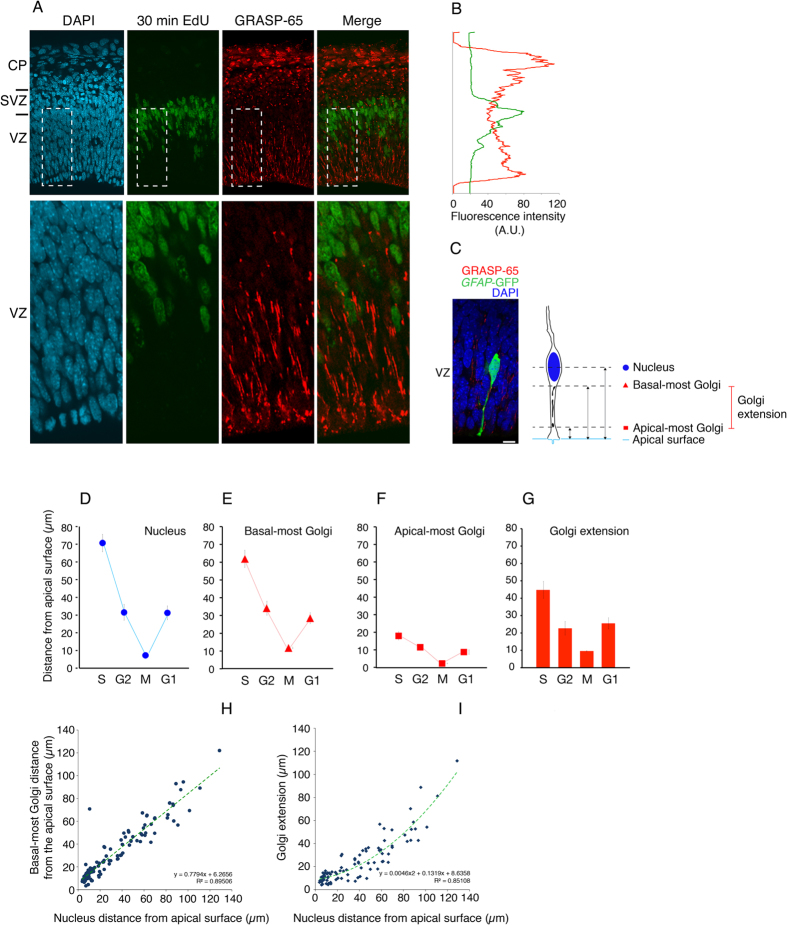
aRG Golgi apparatus during INM. (**A**,**B**) E13.5 wt mouse embryos were pulse-labeled for 30 min with EdU followed by fixation. (**A**) Dorsolateral telencephalon (immuno)stained for GRASP-65 (red) and EdU (green) combined with DAPI staining (cyan). Images are 1-μm optical sections. Scale bar, 20 μm. (**B**) Quantification across cortical wall (top, pia; bottom, ventricle) of the GRASP-65 and EdU (immuno)fluorescence signals shown in (**A)**. (**C–I**) Brains from E13.5 *GFAP*::GFP transgenic mouse embryos were either fixed (to identify aRG in M-phase and G1), or hemispheres were dissected, pulse-labeled for 30 min with EdU and either fixed (to identify aRG in S-phase) or chased for 60, 90 or 120 min followed by fixation (to identify aRG in G2). Dorsolateral telencephalon was (immuno)stained for GRASP-65, GFP and either EdU (aRG in S, G2) or cyclin D1 (aRG in G1), combined with DAPI staining (to identify aRG in M). (**C**) Example of determining the positions of nucleus (DAPI, blue) and Golgi units (GRASP-65, red) in an individual aRG (GFP, green) (single 1-μm optical section). Triple staining was used to determine the distance from apical surface (blue line) of apical-most Golgi unit (red square), basal-most Golgi unit (red triangle) and center of nucleus (blue circle), and to calculate the extension of the Golgi apparatus (see diagram on right). Scale bar, 10 μm. (**D–G**) Distance from apical plasma membrane of aRG nucleus (**D**), basal-most Golgi unit (**E**) and apical-most Golgi unit (**F**), and extension of the Golgi apparatus within the aRG apical process (**G**). Data are the mean of 23 (S, G2), 48 (M) and 42 (G1) individual aRG; error bars indicate SEM. aRG in G2 are the mean of 60, 90 and 120 min of chase. (**H,I**) Correlation between the distance, from apical plasma membrane, of nucleus and either basal-most Golgi unit (**H**) or the Golgi extension (**I**) in 136 identified aRG. Linear correlation in (**H)** (y = 0.7794x + 6.2656, R^2^ = 0.89506), non-linear (second order polynomial) correlation in (**I**) (y = 0.0046 × ^2^ + 0.1319x + 8.6358, R^2^ = 0.85108) (dashed green lines).

The observations that in the single identified aRG studied so far, the Golgi apparatus was found to be located lateral to the nucleus and between the nucleus and the apical membrane (Figs 1 and 2) have implications regarding the overall pattern of Golgi units in the cortical wall. Specifically, one may expect that essentially all aRG Golgi units, that is, the radially oriented GRASP-65–immunoreactive structures in the VZ (Fig. 1E), are located apical to the basal-most position of aRG nuclei. aRG nuclei, in the course of undergoing INM in the VZ, reach this position in S-phase. We therefore identified aRG nuclei in S-phase by a short pulse-labeling with the thymidine analog EdU, and compared the distribution of the GRASP-65–immunoreactive structures in the VZ with the position of these nuclei. Indeed, essentially all aRG Golgi units were found to be located apical to the EdU-positive nuclei in the VZ (Fig. 3A,B).

The question arising from these observations is whether the distribution of the Golgi units within the apical process of aRG, that is, the process extending from the S-phase nucleus in the apical direction to the ventricular surface and spanning most of the VZ, is affected by INM. Specifically, will the aRG nucleus pass Golgi units as it traverses the VZ during G2 towards the ventricular surface for mitosis, such that Golgi units become located basal to the nucleus in late G2? Or will the Golgi units change their location in concert with INM, remaining apical to the nucleus or at most being located lateral to it? To address this question, we again used *GFAP*::GFP mouse embryos and identified single aRG in either S-phase, G2, mitosis, or G1 by using a combination of criteria (see Methods). In these cells, we determined the distance of the nucleus (Fig. 3C,D), of the basal-most Golgi unit as revealed by GRASP-65 immunofluorescence (Fig. 3C,E) and of the apical-most Golgi unit (Fig. 3C,F) to the ventricular surface, followed by calculation of the extension of the Golgi apparatus (Fig. 3C,G). This revealed that irrespective of the cell-cycle phase, the Golgi apparatus in aRG was always located apical to the nucleus (and rarely also lateral to it) and distributed throughout this portion of the apical process (Fig. 3D–F, see also Fig. 1B). Accordingly, the extension of the Golgi apparatus changed during the cell cycle, being greatest in S-phase when the aRG nucleus reaches its basal-most position, and smallest when the aRG nucleus undergoes mitosis near the apical membrane (Fig. 3G). The number of Golgi units per aRG in S-phase ranged from 3 to 6 (average: 4.5 Golgi units per cell, n = 14 cells).

We correlated, in the various aRG analyzed, the position of the basal-most and apical-most Golgi unit, as well as the extension of the Golgi apparatus, to that of the nucleus. This revealed (i) no statistically significant correlation between the position of the apical-most Golgi unit and that of the nucleus (not shown), (ii) a linear correlation between the position of the basal-most Golgi unit and that of the nucleus (Fig. 3H), and (iii) a non-linear correlation between the extension of the Golgi apparatus and the position of the nucleus (Fig. 3I). We conclude from these data that the basal-most rather than apical-most Golgi unit is linked to INM, and that the basal-most Golgi tends to get closer to the nucleus during the late stages of basal-to-apical INM.

### Live imaging of the distribution of the Golgi apparatus in the apical process during interkinetic nuclear migration

**Figure 4:**
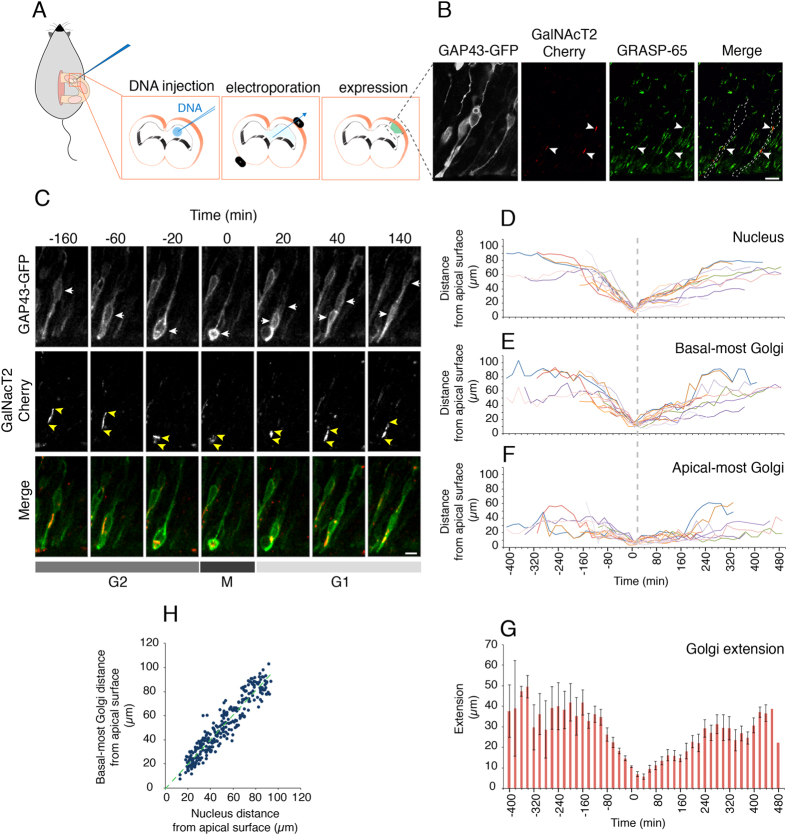
Live imaging of the dynamics of the aRG Golgi apparatus during INM. APs in the VZ of dorsolateral telencephalon of E13.5 wt mouse embryos were co-electroporated *in utero* with plasmids encoding constitutively expressed GAP-43-GFP and GalNAcT2-Cherry; analyses started 24 h later. (**A**) Cartoon of the *in utero* electroporation procedure. (**B**) Expression of GAP-43-GFP (white) and GalNAcT2-Cherry (red) in aRG. Note the colocalization of GalNAcT2-Cherry with Golgi units (arrowheads), as revealed by GRASP-65 immunofluorescence (green). Dashed lines in the right panel indicate the two GAP-43-GFP–expressing aRG containing the indicated Golgi units. Images are stacks of 7 0.8-μm optical sections. Scale bar, 10 μm. (**C**) Still images from live time-lapse imaging of organotypic slices showing an aRG expressing GAP-43-GFP (top row, green in merge (bottom row)) and GalNAcT2-Cherry (middle row, red in merge (bottom row)) and undergoing INM followed by mitosis (0 min time point). Yellow arrows, position of mother aRG nucleus and daughter cell nuclei as revealed by GAP-43-GFP fluorescence; yellow arrowheads, position of apical-most and basal-most Golgi units in mother aRG and apical daughter cell as revealed by GalNAcT2-Cherry fluorescence. Images are stacks of 6 1-μm optical sections. Scale bar, 10 μm. (**D–F**). Live time-lapse imaging traces (20-min intervals) of 14 aRG undergoing INM followed by mitosis (set to 0 min time point, dashed line) and of one of their daughter cells (usually the apical one), showing the distance from the apical plasma membrane of the nucleus as revealed by GAP-43-GFP fluorescence (**D**) and of the basal-most (**E**) and apical-most (**F**) Golgi units as revealed by GalNAcT2-Cherry fluorescence. (**G**) Extension of the Golgi apparatus (from apical-most to basal-most unit) during INM and mitosis in 14 aRG and one of their daughter cells, calculated from the live time-lapse imaging traces shown in (**E**,**F**). Data are the mean ± SEM. (**H**) Correlation between the distance of the basal-most Golgi unit from the apical plasma membrane and the distance of the nucleus from the apical plasma membrane in 578 aRG. Note the linear correlation (y = 1.0159x – 0.4237, R^2^ = 0.8893) (dashed green line).

To corroborate the link between the changes in Golgi apparatus extension in the apical process and INM, and to gain further insights into the dynamics of the aRG Golgi apparatus during INM, we performed live imaging of neocortical aRG in organotypic telencephalic slices in culture^26^. To this end, embryos were *in utero* electroporated (Fig. 4A) at E13.5 with plasmids encoding the membrane-targeting domain of GAP43 fused to GFP (GAP43-GFP) and GalNacT-Cherry, in order to visualize the plasma membrane and the Golgi apparatus of transfected aRG, respectively. Indeed, at E14.5, exogenous GalNacT-Cherry was found to localize to the Golgi apparatus, as demonstrated by the overlap between GalNacT-Cherry fluorescence and endogenous GRASP-65 immunostaining (Fig. 4B). We therefore reasoned that it was appropriate to use exogenous GalNacT-Cherry for studying Golgi apparatus dynamics in aRG by live imaging of organotypic telencephalic slices.

Tracking INM (as revealed by the shape of the cell) by live imaging starting at E14.5, we observed that when the aRG nucleus was present in its basal-most location, the Golgi apparatus showed its greatest extension within the apical process (Fig. 4C–G). During G2, the apical migration of the nucleus towards the ventricular surface was followed by a decrease in the extension of the Golgi apparatus as the proportion of the apical process between the nucleus and the apical plasma membrane became progressively smaller and increasingly curtailed the space available for the Golgi units. These units seemed to progressively become more round and compact (Fig. 4C–G). At mitosis, the Golgi units disassembled into smaller fragments distributed in the now apically located aRG cell body (Fig. 4C), consistent with previous findings with cells in monolayer culture^15^,^27^,^28^. During G1, when the nucleus migrated basally, the Golgi apparatus was composed again of fewer, but larger units, which extended again as the proportion of the apical process between the apical plasma membrane and the nucleus increased and provided progressively more space for the Golgi units (Fig. 4C–G). In line with these observations on Golgi apparatus extension, the basal-most Golgi unit exhibited greater changes in position within the apical process during INM than the apical-most unit (Fig. 4E,F). Upon correlating the position of the basal-most and apical-most Golgi unit as well as the extension of the Golgi apparatus to that of the nucleus as observed by live imaging, we made the same observations (Fig. 4G) as described above (Fig. 3H,I) for the analyses of single aRG in fixed tissue. These live imaging observations therefore corroborated and extended the findings made with fixed tissue (Fig. 3).

### The Golgi apparatus of apical radial glia is not pericentrosomal

**Figure 5:**
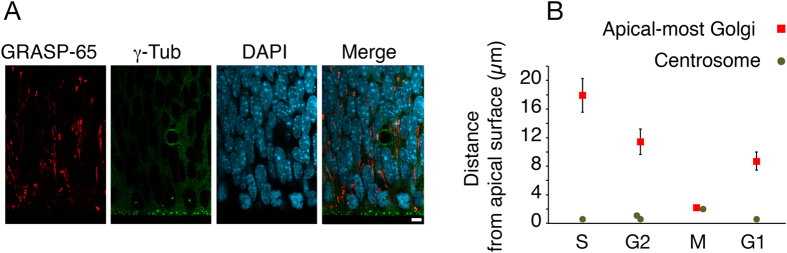
The aRG Golgi apparatus is not pericentrosomal. (**A**) Double immunofluorescence of the VZ of dorsolateral telencephalon of E13.5 wt mouse embryos for the Golgi marker GRASP-65 (red) and the centrosome marker γ-tubulin (green), combined with DAPI staining (cyan). Images are single 1-μm optical sections. Scale bar, 10 μm. (**B**) Dorsolateral telencephalon of E13.5 *GFAP*::GFP transgenic mouse embryos was immunostained for GRASP-65, γ-tubulin and GFP, combined with DAP staining. The distance from the apical plasma membrane of the centrosome (green circles) and the apical-most Golgi unit (red squares) in individual aRG, as revealed by GFP immunofluorescence, was determined from series of optical sections. aRG in specific phases of the cell cycle were identified as described in the legend to Fig. 3C–I. Data for the apical-most Golgi are the same as in Fig. 3F and are the mean of 23 (S, G2), 48 (M) and 42 (G1) individual aRG; data for the centrosome are the mean of 38 (S), 55 (G1, G2) and 20 (M) individual aRG; error bars indicate SEM.

In mammalian cells, the Golgi apparatus is generally thought to be located near the nucleus and close to the centrosome^13^,^29^. By contrast, the data we have presented so far indicate that in aRG, the Golgi apparatus is localized between the nucleus and the centrosome(s), which is/are known to be tethered to the apical plasma membrane, reflecting the presence of a primary cilium protruding from this membrane into the ventricular lumen^30^,^31^. These data already imply that the Golgi apparatus as a whole is not pericentrosomal in aRG. We corroborated this conclusion by immunostaining mouse E13.5 neocortex for GRASP-65 and γ-tubulin, a centrosomal marker (Fig. 5A, see also EM in Fig. 2). This confirmed that, in the VZ, the centrosomes are concentrated at the ventricular surface and the Golgi apparatus did not appear to extend all the way to the centrosome(s) (Fig. 5A).

However, these data did not strictly exclude the possibility that in aRG, the apical-most Golgi unit is located in the immediate vicinity of the centrosome(s) (i.e., pericentrosomal). To directly investigate this issue, we again used E13.5 *GFAP*::GFP mouse embryos to identify single aRG in either S-phase, G2, mitosis, or G1 (see above and Methods), and compared the localization of the apical-most Golgi unit (see Fig. 3F) to that of the centrosome(s) (Fig. 5B). This revealed that in the interphase aRGs examined, the apical-most Golgi unit was always >8 μm away from the centrosome (Fig. 5B). The greatest distance between the apical-most Golgi unit and the centrosome(s) was observed in S-phase (18 μm, Fig. 5B). These data demonstrate that none of the Golgi units in interphase aRG are pericentrosomal.

### A non-pericentrosomal Golgi apparatus is an evolutionary conserved feature of columnar epithelia

**Figure 6:**
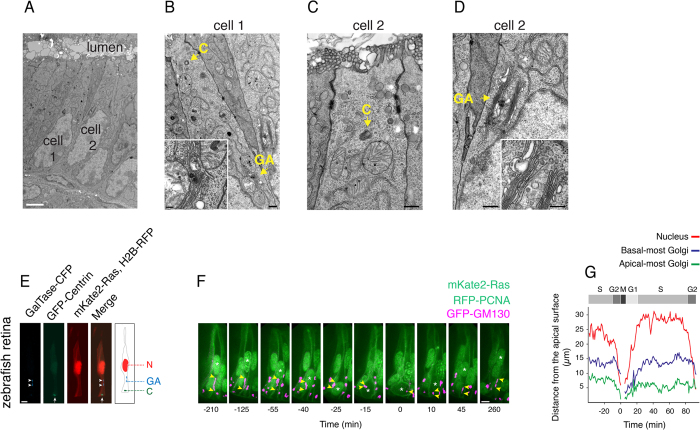
A non-pericentrosomal localization and an INM-linked extension of the Golgi apparatus are evolutionary conserved features of columnar epithelial cells. (**A–D**) The Golgi apparatus in cells of the simple columnar epithelium of adult mouse duodenal mucosa. (**A**) Transmission electron micrograph. Cell 1and cell 2 are shown at higher magnification in (**B**) and (**C**,**D**), respectively. Scale bar, 2 μm. **(B–D**) Higher magnification transmission electron micrographs showing the Golgi apparatus (GA) and centrosome (**C**) of cell 1 (**B**) and cell 2 (**C**,**D**) indicated in (**A**). Insets in (**B**,**D**), Golgi apparatus. Scale bars, 200 nm; insets, 100 nm. (**E–G**) Dynamics of the Golgi apparatus in pseudostratified columnar epithelial cells of developing zebrafish retina. (**E**) Confocal image (stack of 5 1-μm optical sections) showing the localization of the Golgi apparatus (CFP-tagged beta-galactosyltransferase, GalTase–CFP, cyan, arrowheads) and centrosome (GFP-centrin, green, arrows) in a retinal neuroepithelial cell. The plasma membrane and nucleus are revealed by expression of mKate2-ras (red) and histone H2B-RFP (red), respectively. Diagram: localization of the Golgi (GA), apical centrosome (**C**) and nucleus (N) as reconstructed from single optical sections. Scale bar, 5 μm. (**F**) Still images (stacks of 5–10 1-μm optical sections) from live time-lapse imaging (5-min intervals) showing the localization and extension of the Golgi apparatus in a retinal neuroepithelial cell and one of its daughter cells as revealed by expression of GFP-GM130 (magenta); apical-most and basal-most extension of the Golgi apparatus is indicated by arrowheads. The position of the nucleus and the progression through the cell cycle are revealed by expression of RFP-PCNA (green; mother cell S-to-G2, punctate-to-diffuse nuclear pattern; daughter cell G1-to-S, diffuse-to-punctate nuclear pattern). The plasma membrane is revealed by expression of mKate2-ras (green). The mother cell undergoes INM followed by mitosis (set to 0 min time point), and the nuclei of the daughter cells migrate basally (nuclei are indicated by asterisks). Scale bar, 5 μm. (**G**) Live time-lapse imaging traces (5-min intervals) of the mother neuroepithelial cell and its apical daughter cell shown in (**F**). Cell cycle phases were deduced from the nuclear PCNA pattern.

We investigated whether the non-pericentrosomal Golgi apparatus observed in mouse aRG is a feature conserved in other columnar epithelial cells and in other vertebrates. With regard to other columnar epithelial cells in the same species, we analyzed the adult mouse duodenal mucosa. Serial sectioning EM analysis demonstrated that also in these epithelial cells (which appeared to lack primary cilia, Fig. 6A), the centrosomes are located near the lumenal surface (on average within 1.5 μm; Fig. 6B,C), similar to the situation in aRG. In contrast, the Golgi apparatus was found to be located in the vicinity of the nucleus (on average within 1.7 μm) and away from the centrosome (on average >5 μm; Fig. 6B,D). Hence, a non-pericentrosomal localization of the Golgi apparatus appears to be a common feature of mouse columnar epithelial cells.

To examine columnar epithelial cells in other, non-mammalian vertebrates, we analyzed the Golgi apparatus in the developing zebrafish retina at a stage when it consists of a pseudostratified columnar epithelium, formed by neuroepithelial cells highly related to, although not as elongated as, mouse aRGs. Figure 6E shows an example of a zebrafish retinal neuroepithelial cell in which the Golgi apparatus (visualized by expression of GalTase–CFP) was found to be non-pericentrosomal, as it was localized half-way between the nucleus (seen at mid-position along the cell’s apical-basal axis) and the apical centrosome (visualized by expression of GFP-centrin). Furthermore, and similar to what we observed in mouse aRG (Fig. 2B), the long axis of the Golgi cisternae was oriented parallel to the apical-basal axis of the cell, as also revealed by visualizing the orientation of microtubules using expression of the plus-end binding protein EB3 fused to mKate2 (Supplementary Figure S2, A). When the nucleus (as a result of INM) was near the apical plasma membrane, and hence the inter-centrosome-nucleus space curtailed, a portion of the Golgi apparatus could be observed in the vicinity of the centrosome (data not shown).

We then used live imaging to study the dynamics of the Golgi apparatus in zebrafish neuroepithelial cells, visualized by expression of GFP-GM130, in relation to the different cell cycle phases, visualized by expression of RFP-PCNA^32^. Consistent with our findings with mouse aRG (Fig. 4), the extension of the Golgi apparatus decreased as the nucleus approached the apical plasma membrane in G2, and increased again as the nucleus moved from apical to basal in G1 (Fig. 6F,G). We conclude that similar to mouse aRG, the Golgi apparatus exhibits a largely non-pericentrosomal localization and cell cycle-dependent dynamics in concert with nuclear migration also in non-mammalian columnar epithelial cells, indicating that these features are evolutionary conserved.

We took advantage of the zebrafish system to gain insight into the machinery mediating the localization of the Golgi apparatus, focusing our attention on the role of microtubules^33^. We found that microtubule depolymerization via stathmin and inhibition of dynein motors leads to the fragmentation of the Golgi apparatus and its dispersal in the basal direction (Supplementary Figure S2, B–D). In contrast, inhibition of the kinesin-1 motor had the opposite effect, leading to the concentration of the Golgi apparatus just beneath the apical plasma membrane (Supplementary Figure S2, B, E). These data indicate that although in vertebrate columnar epithelial cells the Golgi apparatus is not pericentrosomal, a microtubule-based machinery mediates its subcellular localization, similar to non-epithelial cells in which the Golgi apparatus is pericentrosomal.

### The Golgi apparatus undergoes basal translocation during basal progenitor delamination

**Figure 7:**
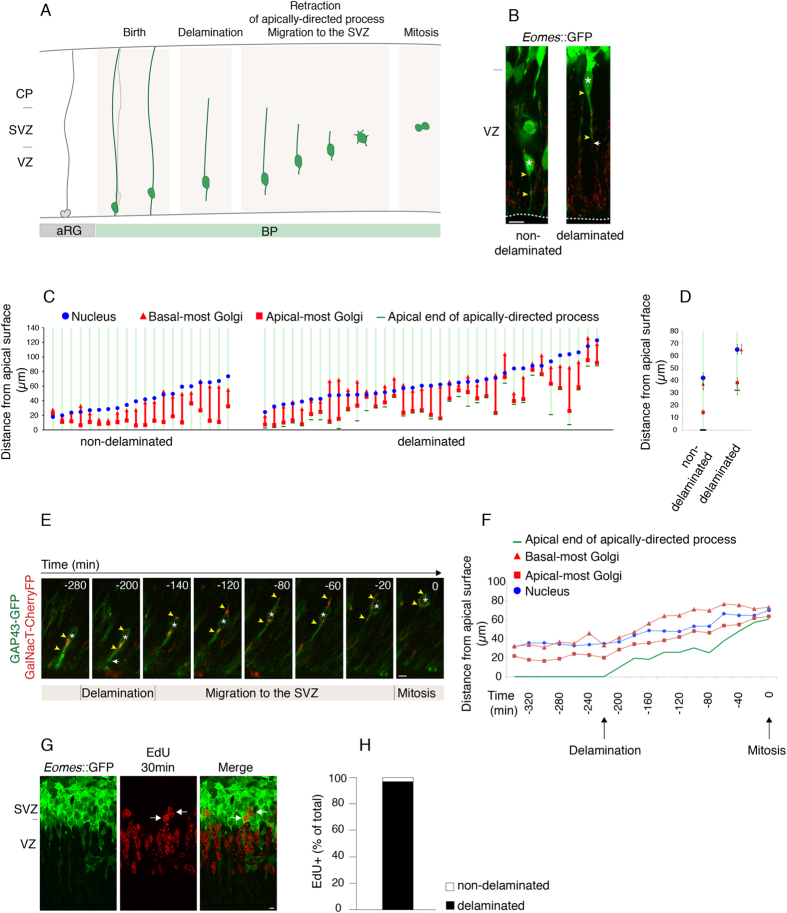
Subcellular localization and extension of the Golgi apparatus in newborn delaminating BPs. Analysis of the Golgi apparatus in newborn, delaminating BPs (**A**) in dorsolateral telencephalon of E13.5 *Eomes*::GFP transgenic mouse embryos (**B–D, G,H**) and of E14.5 wt mouse embryos co-electroporated *in utero* at E13.5 with plasmids encoding GAP-43-GFP and GalNAcT2-Cherry (**E,F**). (**A**) Cartoon illustrating major features of basal intermediate progenitor (bIP) delamination. (**B**) Subcellular localization of the Golgi apparatus (GRASP-65 immunofluorescence, red) in a non-delaminated (left) and delaminated (right) bIP (*Eomes*::GFP, green). Asterisks, nucleus; arrowheads, basal-most and apical-most Golgi unit; arrow, retracting apically directed process. Dashed lines, ventricular surface. Images are stacks of 5 (left) and 6 (right) 0.9-μm optical sections. Scale bar, 20 μm. (**C**) Distance from apical surface of the nucleus (blue circles), basal-most (red triangles) and apical-most (red squares) Golgi units, and apical end of apically directed process (green horizontal bars) in individual (light green vertical lines) BPs with (non-delaminated, left) and without (delaminated, right) apical contact. (**D**) Mean of the data shown in (**C**); error bars indicate SEM. (**E**) Still images from live time-lapse imaging (20-min intervals) of organotypic slices showing a newborn bIP expressing GAP-43-GFP (green) and GalNAcT2-Cherry (red) that undergoes delamination from the ventricular surface followed by migration to the SVZ and mitosis. Asterisks, nucleus; yellow arrowheads, apical-most and basal-most Golgi units; white arrow, apical end of the retracting apically directed process. Images are stacks of 3 1-μm optical sections. Scale bar, 10 μm. (**F**) Live time-lapse imaging traces of the newborn delaminating bIP shown in (**E**). (**G**) E13.5 *Eomes*::GFP mice were pulse-labeled for 30 min with EdU followed by fixation. Dorsolateral telencephalon was (immuno)stained for GFP (to identify individual BPs, green) and EdU (to identify BPs in S-phase, red). Arrows, EdU-positive BPs. Images are single 1-μm optical sections. Scale bar, 5 μm. (**H**) Percentage of EdU-positive BPs in S-phase, labeled and identified as in (**G**), lacking (delaminated, black) or still exhibiting (non-delaminated, white) apical contact, as reconstructed from the series of optical sections of *Eomes*::GFP immunofluorescence.

Next, we investigated the subcellular localization of the Golgi apparatus during the delamination of BPs, that is, upon loss of apical cell polarity. To specifically reveal the morphology of single BPs, we made use of the *Eomes*::GFP mouse line, in which a cytosolic GFP is under the control of the promoter of *Eomes*, which encodes Tbr2, a BP marker^34^. We compared newborn BPs that had not yet delaminated, i.e. that still contacted the ventricle and were embedded in the apical adherens junction belt (Fig. 7A,B left), with delaminated BPs (Figure A,B right). This revealed that in newborn, non-delaminated BPs, the Golgi apparatus in most cases was distributed between the nucleus and the apical plasma membrane (Fig. 7C,D left), similar to its distribution in aRG. In contrast, in delaminated BPs, the basal-most Golgi unit in many cases was observed in the process basal to the nucleus, and in several instances the distance between the apical-most Golgi unit and the apical end of the retracting apically-directed process was >10 μm (Fig. 7C,D right), that is, greater than in aRG in G1 (see Fig. 5B).

These features of a translocation of the Golgi apparatus in the basal direction were also observed when the overall process of BP delamination, including the basal migration of the nucleus and the retraction of the apically-directed process, were analyzed by live imaging (Fig. 7E,F). In addition, live imaging revealed that the basal-most Golgi moves past the nucleus and into the process basal to it, rapidly after the newborn BP has actually delaminated from the ventricle and apical adherens junction belt.

The apical-to-basal migration of the nucleus of delaminating BPs is thought to occur during G1^35^. As judged from the position of the nucleus relative to the apical-basal dimension of the VZ, the actual delamination step of BPs from the ventricle and apical adherens junction belt can take place early (Fig. 7C right, left cell) or late (Fig. 7C left, right cell) in G1. To further investigate the delamination step in relation to cell cycle progression, we pulse-labeled E13.5 *Eomes*::GFP mouse embryos for 30 min with EdU to identify BPs in S-phase (Fig. 7G). Almost all (98%) BPs in S-phase were found to lack apical contact (Fig. 7H), indicating that the delamination of BPs from the ventricle and apical adherens junction belt virtually always occurs in G1.

### The Golgi apparatus becomes pericentrosomal in delaminated basal progenitors

**Figure 8:**
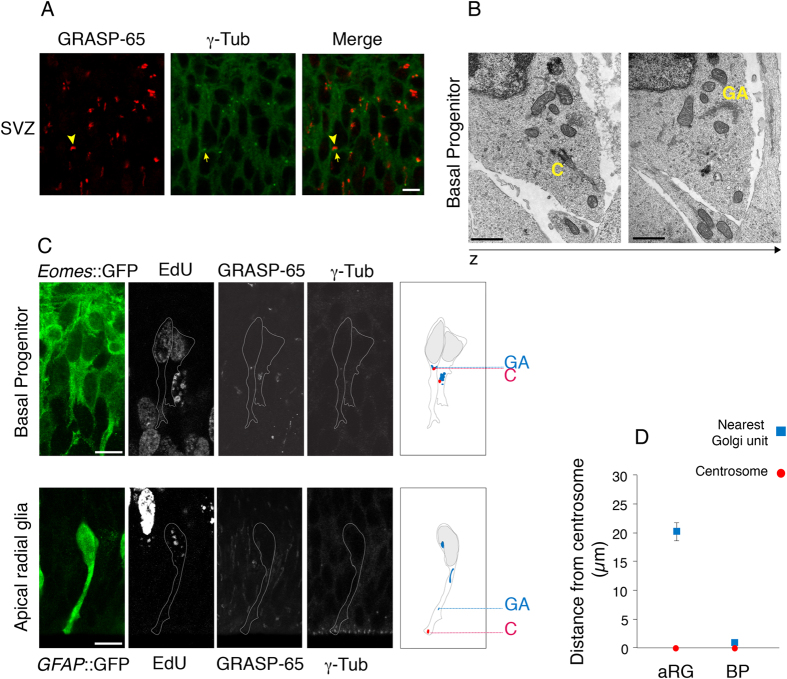
The Golgi apparatus becomes pericentrosomal in delaminated BPs. (**A**) Subcellular localization of the Golgi apparatus, as revealed by GRASP-65 immunofluorescence (red), and centrosomes, as revealed by the punctate γ-tubulin immunofluorescence (green), in the SVZ of E13.5 wt mouse dorsolateral telencephalon. An example showing the proximity of the Golgi apparatus (arrowheads) and the centrosome (arrows) is indicated. Images are single 1-μm optical section. Scale bar, 10 μm. (**B**) Adjacent transmission electron micrographs of a presumptive bIP in the SVZ of E13.5 wt mouse dorsolateral telencephalon. Left section shows the centrosome (**C**) constituting the basal body of a primary cilium; right section shows the Golgi apparatus (GA) Scale bars, 1 μm. (**C**) E13.5 *Eomes*::GFP (top row) or *GFAP*::GFP (bottom row) transgenic mice were pulse-labeled for 30 min with EdU followed by fixation. Dorsolateral telencephalon was (immuno)stained for GFP (to identify individual aRG (bottom) and BPs (top), green), GRASP-65 (to reveal the position of the Golgi units), γ-tubulin (to identify centrosomes), and EdU (to identify progenitors in S-phase). Diagrams on the right show the localization of Golgi units (blue), with the one nearest to the centrosome (**C**) indicated (GA), in the respective S-phase cells, as reconstructed from the series of optical sections. Images are stacks of 3 (top) and 2 (bottom) 0.9-μm optical sections. Scale bars, 10 μm. (**D**) Distance between the position of the centrosome (set to zero, red circles) and the nearest Golgi unit (blue squares) in S-phase aRG and BPs, labelled, (immuno)stained and analyzed as in (**C**). Data are the mean of 38 aRG and 20 BPs; error bars indicate SEM.

In light of the basal translocation of the Golgi apparatus during BP delamination, we analyzed the spatial relationship between the Golgi apparatus and the centrosome in fully delaminated BPs in the SVZ of E13.5 mouse neocortex. Comparison of GRASP-65 and γ-tubulin immunofluorescence revealed that, in contrast to the aRG in the VZ (see Fig. 5A), the Golgi apparatus in cells in the SVZ typically is next to the centrosome(s), which in turn is (are) usually located in the vicinity of the nucleus (Fig. 8A). This finding was further corroborated by EM (Fig. 8B).

To determine the spatial relationship between the Golgi apparatus and the centrosome at the single cell level, we measured the distance between the γ-tubulin–stained centrosome(s) and the nearest GRASP-65–positive Golgi unit in 30-min EdU pulse-labeled, *Eomes*::GFP-positive cells in the SVZ of E13.5 mouse neocortex, that is, in fully delaminated BPs in S-phase (Fig. 8C top). This phase of the cell cycle was chosen as by then, virtually all BPs have delaminated from the ventricle and apical adherens junction belt (see Fig. 7H). In contrast to *GFAP*::GFP-positive, S-phase aRG in the VZ, in which the nearest Golgi unit was on average 20 μm distant from the apically located centrosome (Fig. 8C bottom, D, confirming the data of Fig. 5B), Golgi units were always found in the immediate vicinity (<1 μm) of the perinuclearly located centrosomes of fully delaminated, S-phase BPs in the SVZ (Fig. 8A top, D).

Taken together, these data show that concomitant with the transition from a bipolar epithelial stem cell exhibiting apical-basal polarity to a delaminated progenitor lacking apical cell polarity, the Golgi apparatus undergoes a fundamental reorganization – from non-pericentrosomal and non-perinuclear to pericentrosomal and perinuclear.

### Golgi apparatus disassembly and reassembly during mitosis differs between apical and basal progenitors

In light of the difference between aRG and BPs with regard to the Golgi apparatus–centrosome relationship during interphase, and given that centrosomes function as spindle poles during mitosis, we asked whether differences exist between aRG and BPs in mitosis with regard to Golgi apparatus dynamics and mitotic spindle association^16^,^36^,^37^,^38^,^39^,^40^,^41^.

**Figure 9:**
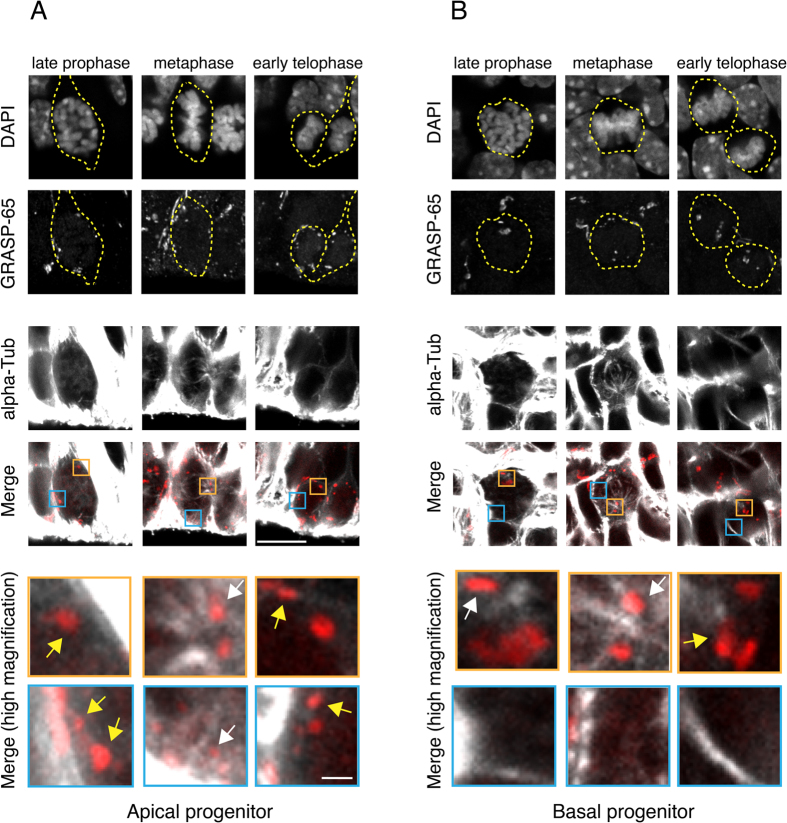
Golgi apparatus in mitotic neural progenitor. Subcellular localization of the Golgi apparatus, as revealed by GRASP-65 immunofluorescence, in mitotic APs (**A**) and BPs (**B**) of E14.5 wt mouse dorsolateral telencephalon. Cell contours (yellow dashed lines) were deduced from phalloidin staining (not shown). The mitotic phase was determined based on the state of DNA condensation and organization (DAPI staining), and the mitotic spindle is revealed by immunofluorescence for α-tubulin. Blue and orange boxes: high magnification of areas highlighted in the Merge, showing the proximity of Golgi units to the mitotic spindle (white arrows) or their localization near the cell periphery (yellow arrows). Scale bar, low magnification 5 μm, high magnification 0.5 μm.

During prophase, the Golgi apparatus of APs appeared to undergo fragmentation, as revealed by the increased number and reduced size of GRASP-65 immunoreactive structures (Fig. 9A), and the Golgi remnants, consisting of partially stacked cisternae, were distributed at the cell periphery and only partially observed in the vicinity of the spindle poles, revealed by α-tubulin immunofluorescence (Fig. 9A). In contrast, the Golgi apparatus of BPs in prophase appeared more compact, and most of it was observed in close proximity to the spindle poles (Fig. 9B). This Golgi apparatus–spindle pole association persisted in metaphase BPs (Fig. 9B, Supplementary Figure S3). In metaphase APs, in addition to some spindle pole-associated Golgi structures, the Golgi structures at the cell periphery were now seen in proximity to astral microtubules, reflecting the maturation of the mitotic spindle at this stage of mitosis (Fig. 9A, Supplementary Figure S3).

At telophase, when the Golgi apparatus is known to re-assemble^13^,^38^, the Golgi structures in APs were again observed to be distributed at the cell periphery (Fig. 9A). In contrast, in telophase BPs, the Golgi structures were largely observed near the midbody bridge (Fig. 9B), which harbors the remnants of the mitotic spindle^42^. Taken together, these data show that the two main classes of neural progenitor cells in the developing mouse neocortex, aRG and BPs, differ with regard to the Golgi apparatus–centrosome relationship not only in interphase, but also during mitosis.

At mid-neurogenesis, aRG mostly undergo asymmetric self-renewing division, yielding an aRG and a BP, whereas BPs typically undergo symmetric self-consuming division, yielding two neurons^7^,^8^,^9^,^10^. We investigated whether there was a similar asymmetry vs. symmetry in the partitioning of the Golgi units to the two daughter cells arising from aRG vs. BP division, respectively. However, consistent with the GRASP-65 immunofluorescence data (Fig. 9A,B), SBF-SEM revealed a largely similar distribution of Golgi units between the nascent daughter cells for both AP and BP divisions (Supplementary Figure S4, A,C). This was corroborated by quantification of the distribution of GRASP-65 immunoreactive structures between the nascent daughter cells of anaphase/telophase APs and BPs, which revealed a partitioning ranging from 50:50% to 60:40% in the vast majority of cases for both, AP and BP divisions (Supplementary Figure S4, B, D). We conclude that aRG asymmetric division vs. BP symmetric division has no counterpart with regard to the partitioning of Golgi units during cytokinesis. This conclusion is in line with the notion that at the subcellular level, the hallmark of aRG asymmetric division is the asymmetric inheritance by the aRG daughter cell of the basal process, which lacks Golgi structures (Figs 1 and 2).

### In contrast to the Golgi apparatus, the endoplasmic reticulum of aRG is present not only in the apical but also basal process

**Figure 10:**
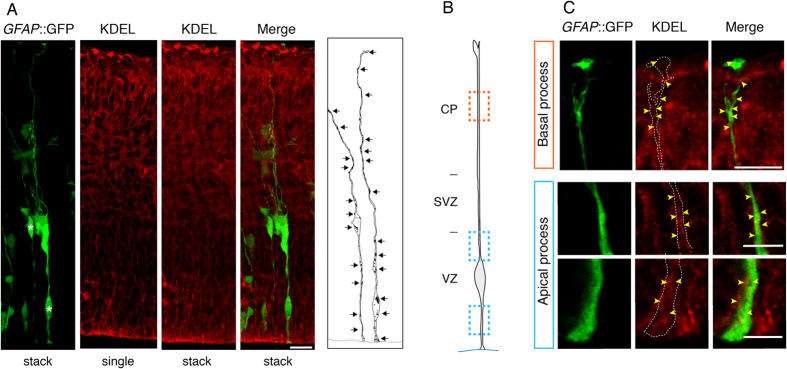
The ER is present in both apical and basal process of aRGs. (**A**) Subcellular localization of the ER, revealed by immunofluorescence for KDEL-containing ER-resident proteins (red), in aRGs in dorsolateral telencephalon of an E14.5 *GFAP*::GFP transgenic mouse embryo, identified by GFP immunofluorescence (green). Images are either stacks of six 0.6-μm single optical sections, or one single optical section, as indicated. Diagram on the right shows the localization of endoplasmic reticulum (arrows) in the two single aRGs the nuclei of which are indicated by asterisks in the left panel, as reconstructed from the series of optical sections. Note that the ER staining is more diffuse in the stack than in the single optical section, and appears non-continuous. The latter may reflect local inhomogeneities, within the ER, of KDEL-containing proteins that are related to the specific aRG cell architecture in tissue (see also the pattern of Sec61 and Sec16 immunofluorescence, Figure S5). Scale bar, 20 μm. (**B**) Cartoon of an aRG illustrating the regions of the apical and basal process subjected to immunofluorescence analysis in (**C**). Blue boxes, apical process in the VZ apical and basal to the nucleus; orange box, basal process in the CP. (**C**) High magnification of portions of the basal process (top row), the apical process above the nucleus (middle row) and the apical process below the nucleus (bottom row) of a single aRG in dorsolateral telencephalon of an E14.5 *GFAP*::GFP transgenic mouse embryo, identified by GFP immunofluorescence (green), showing the presence of ER (arrowheads) as revealed by immunofluorescence for KDEL-containing ER-resident proteins (red). Dashed lines indicate the apical or basal process of the aRG analyzed. Images are single 0.6-μm optical sections. Scale bars, 2 μm.

Next, we explored the functional significance of the non-canonical organization of the Golgi apparatus in aRG, notably its confinement to their apical process. Given that the Golgi apparatus is known to function as a major transit and sorting station for membrane traffic in the secretory pathway, we asked whether in aRG, the rough endoplasmic reticulum (ER), which delivers newly synthesized secretory, lysosomal and membrane proteins to the Golgi apparatus, exhibited a similar, apical process-confined subcellular localization as the Golgi apparatus. However, analysis of E14.5 *GFAP::*GFP–positive aRG by immunofluorescence for KDEL-containing ER-resident proteins (Fig. 10) including protein disulfide isomerase (not shown), and for Sec61 (translocon; Supplementary Figure S5, A,B) and Sec16 (ER exit sites (ERES) marker; Supplementary Figure S5, A,C), a rough ER transmembrane and peripheral membrane protein, respectively^43^,^44^,^45^, revealed that unlike the Golgi apparatus, the (rough) ER of aRG is present not only in their apical, but also in the basal process, extending from the apical endfoot at the ventricle all along the apical-basal axis of the cell to the basal endfoot at the basal lamina. Transmission EM corroborated the presence of rough ER in both the apical and basal process of aRG (Supplementary Figure S6).

### The plasma membrane of the aRG basal process lacks Golgi-derived membrane constituents

The findings that in aRG the rough ER and ERES (the sites of COPII-mediated vesicle budding) are present in both the apical and basal process whereas the Golgi apparatus is confined to the apical process, raise important questions regarding the mode of delivery of newly synthesized membrane constituents to the basal process plasma membrane. Specifically, are all these membrane constituents transported from the ER to the plasma membrane via the Golgi apparatus? Or do newly synthesized membrane constituents destined to the basal process plasma membrane bypass the Golgi apparatus, being delivered via an unconventional vesicular secretory pathway^46^,^47^,^48^,^49^? Addressing these questions is particularly relevant in light of the facts that (i) the basal process of aRG is significantly longer than the apical one, notably in large-brain species such as human; (ii) the basal process of aRG increases in length during cortical development; and (iii) newly generated aRG may re-grow a basal process. All of these facts imply that the supply of newly synthesized membrane constituents to the aRG basal process plasma membrane must be substantial.

The processing of N-linked glycans on rough ER-translated membrane proteins offers a means to addressing these questions. During passage through the Golgi apparatus, these N-linked glycans are converted from the ER-specific high-mannose type to the complex type^50^. Such conversion does not take place if the Golgi apparatus is bypassed when delivery to the plasma membrane occurs via an unconventional vesicular secretory pathway. High-mannose type and complex type N-linked glycans can be specifically detected by the lectins concanavalin A (ConA) and wheat-germ agglutinin (WGA), respectively^50^.

We therefore explored the possibility of using WGA vs. ConA cell-surface staining to detect possible differences in the abundance of Golgi-delivered vs. non-Golgi-delivered plasma membrane constituents in the apical vs. basal process of aRG, respectively. Indeed, using either fluorescently labeled ConA or WGA on paraformaldehyde-fixed, non-detergent-treated tissue sections, we observed staining across the entire apical-basal axis of the cortical wall that outlined the surface of cells, including the ventricular surface (Supplementary Figure S7). In addition, for some cells, staining of the nuclear envelope was observed with ConA, but not WGA (Supplementary Figure S7). Reflecting some membrane permeabilization due to the paraformaldehyde fixation, this documented the specificity of the ConA staining for the ER-characteristic high-mannose type of N-linked glycans.

**Figure 11:**
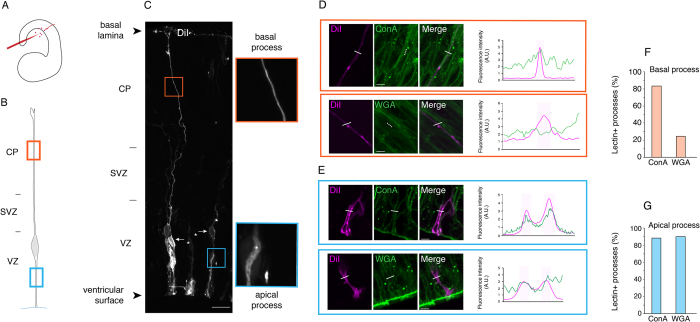
The plasma membrane of the aRG basal process lacks Golgi-modified glycans. E14.5-16.5 wt mouse cerebral hemispheres were labeled with DiI applied to the ventricular surface, fixed and cell surface-stained with ConA-A488 or WGA-A488 (**D**–**G**). (**A**) Cartoon illustrating DiI labeling. (**B**) Cartoon of a DiI-labeled aRG illustrating the region of the apical and basal process subjected to lectin staining and quantification in (**D**–**G**). Blue box, apical process in VZ apical to nucleus; orange box, basal process in CP. (**C**) DiI fluorescence showing single labeled aRGs including their apical process (blue boxes) and basal process (orange boxes). Arrows with asterisk, nucleus of the two indicated aRGs. The image is a stack of 20 0.6-μm single optical sections. Scale bar, 20 μm. (**D,E**) ConA (green, (**D**) top row, (**E**) top row) and WGA (green, (**D**) bottom row, (**E**) bottom row) fluorescence of a DiI-labeled basal (**D**) and apical (**E**) process (magenta). Images are 0.6-μm single optical sections. Solid white lines: orientation of the scans to measure fluorescence intensity, as shown in the line plots on right: magenta lines, DiI fluorescence; green lines, ConA or WGA fluorescence; A.U., arbitrary units. The scanned DiI-labeled basal process (**D**) is indicated by a dotted white line positioned immediately adjacent to the process. Magenta rectangular areas in line plots indicate the width of the process in the scan; in the case of the apical process (**E**) the plasma membrane on either side of the process can be distinguished. Note the presence of both, ConA-positive and WGA-positive constituents in the plasma membrane of the apical process (**E**), and the presence of ConA-positive, but the lack of WGA-positive, constituents in the plasma membrane of the basal process (**D**). Scale bars, 5 μm. (**F,G**) 84 and 41 basal (**F**) and 43 and 107 apical (**G**) processes were analyzed as in (**D**,**E**), respectively, for the occurrence of ConA or WGA cell surface staining. The colocalization of a peak of lectin staining with the peak of DiI labeling in line plots was scored as positive lectin staining. The percentage of lectin + processes is shown.

To gain single-cell resolution and to identify the apical and basal process of single aRG in order to assess the occurrence of ER-type vs. Golgi-type N-linked glycans on its plasma membrane, we combined the lectin cell surface staining with labeling using DiI, a lipophilic dye that diffuses in the plane of the membrane. DiI was sparsely applied to the ventricular surface of E14.5 hemispheres to label the apical plasma membrane of individual neocortical aRG^51^ (Fig. 11A). Given the absence of tight junctions^52^, this allowed the subsequent diffusion of the dye to the basolateral plasma membrane after paraformaldehyde fixation. This enabled us to specifically delineate the morphology of single aRG, including their apical and basal process (Fig. 11B,C).

Analysis of the lectin cell-surface staining of single DiI-labeled apical processes revealed that their plasma membrane almost always contained both, ConA-positive, ER-type and WGA-positive, Golgi-type N-linked glycans (Fig. 11E,G). In striking contrast, the plasma membrane of single DiI-labeled basal processes of aRG traversing the SVZ and CP was found to contain WGA-positive, Golgi-type N-linked glycans only rarely, whereas ConA-positive, ER-type N-linked glycans were observed in the vast majority of cases (Fig. 11D,F). These data suggest that newly synthesized rough ER-derived membrane constituents are delivered to the basal process plasma membrane largely via an unconventional vesicular secretory pathway that bypasses the Golgi apparatus.

## Discussion

We have studied the organization and dynamics of the Golgi apparatus in aRGs, the neural stem cells of the developing neocortex. These cells exhibit extreme features of cell polarity and thus lend themselves to uncover general principles regarding Golgi organization and cell polarity. In addition, the developing neocortex provides the opportunity to study the influence of the loss of cell polarity on the organization of the Golgi apparatus, as aRGs give rise to progenitor daughter cells lacking cell polarity. Our findings call for a revision of current views on the Golgi apparatus with regard to (i) its subcellular localization in polarized epithelial cells, (ii) its dynamics in interphase, (iii) its association with the centrosome, (iv) its reorganization upon the loss of apical cell polarity, and (v) its role in the polarized delivery of membrane constituents to the cell surface. Moreover, our findings provide novel insight into the cell biological basis of neural stem and progenitor cell diversity and tissue architecture in developing neocortex.

### Exclusive occurrence of the Golgi apparatus in the apical but not basal process of aRGs

Unlike the ER, the Golgi apparatus was exclusively found in the apical, but not basal, process of aRGs. This confinement likely reflects the fact that aRGs are derived from neuroepithelial cells. The latter cells lack the basal process that aRGs form to retain contact with the basal lamina as the cell layers basal to the VZ arise. In other words, the apical process of neuroepithelial cells is the only cytoplasmic space available to harbour the Golgi complex, and this feature is presumably conserved as these cells transform into aRGs.

Why does the Golgi apparatus not pass by the nucleus and enter the basal process of aRGs, as for example the ER appears to do? We speculate that one reason may lie in the way the Golgi membrane system is organized. The Golgi is formed by stacked cisternae kept together by Golgi matrix proteins (Golgins, GRASP-65)^53^. These Golgi stacks likely exhibit some stiffness^54^, which in turn may limit their ability to pass through the narrow space between the nuclear envelope and the lateral plasma membrane. In line with this notion, the only situation when Golgi units were found in this narrow space was when the stacks fragmented in late G2.

### Dynamics of the Golgi apparatus during INM

The presumed inability of the stacked Golgi cisternae to pass between the nuclear envelope and the lateral plasma membrane likely also explains, at least in part, why the aRG Golgi apparatus, within the apical process, was always found apical to the nucleus, as corroborated by 3D reconstruction using SBF-SEM. This was particularly remarkable at the end of G2, when the nucleus due to INM had migrated within the apical process towards the apical plasma membrane, curtailing the space for intracellular organelles between this end of the aRG and the nucleus.

Interestingly, when the aRG nucleus migrated towards the basal VZ in G1, the Golgi units did not simply remain clustered in the apical-most cytoplasm, but also translocated basally within the apical process, extending across most of the VZ by S-phase. These observations suggest that the cell cycle-dependent alterations in Golgi apparatus extension that occur concomitant with INM and that were followed by live imaging in the developing mouse neocortex and the zebrafish retina, are not only passive in nature but involve an active machinery, at least during G1. Our experiments in zebrafish suggest that microtubules and microtubule-based motors are part of this machinery.

On a more general note, these observations are, to the best of our knowledge, the first demonstration showing that an intracellular organelle is reorganized to match the movement of the nucleus during INM. Furthermore, this dynamic behavior of the Golgi apparatus in concert with the cell cycle appears to be an evolutionarily conserved feature of pseudostratified epithelia, as it was observed in fish and mammals.

### Absence vs. presence of centrosome association of the Golgi apparatus and neural progenitor cell fate transition

Surprisingly, and contrary to the commonly held view, the Golgi apparatus in interphase aRGs is not pericentrosomal. Conversely, in newborn BPs the Golgi apparatus relocalizes and becomes pericentrosomal by the end of the delamination process. BP delamination involves the loss of apical attachment and apical cell polarity^2^,^7^,^8^,^9^. Hence, it is conceivable that the absence of centrosome association of the aRG Golgi apparatus is linked to the presence of apical features, and the presence of this association in BPs to the loss of these features.

The absence vs. presence of centrosome association of the Golgi apparatus in aRGs vs. BPs, respectively, had a temporal correlate with regard to the timing of Golgi apparatus disassembly and reassembly during mitosis. Specifically, aRG appeared to disassemble the Golgi apparatus earlier and reassemble it later in mitosis than BPs. In other words, the duration of the disassembled state of the Golgi apparatus was longer in aRG than BPs. This may have consequences for cell fate. The disassembling mitotic Golgi apparatus releases several proteins^13^,^15^,^55^,^56^,^57^. Among them, the most notable example is ACBD3, a protein that is released at the onset of mitosis into the cytosol, where it affects cell fate by acting upon the Numb/Notch signaling pathway^58^. The ability of ACBD3 to affect cell fate is therefore confined to a limited temporal window, determined by the disassembly and reassembly of the Golgi apparatus. The longer disassembled state of the Golgi apparatus in mitotic aRGs than BPs may therefore result in a longer exposure of the former cells to Numb/Notch signaling, which in turn may impact on cell fate choice and stemness.

### Golgi apparatus independence of the plasma membrane of the aRG basal process

Our data show that the Golgi apparatus, in contrast to the ER, is strictly confined to the aRG apical process. Of note, the basal process does not contain structures homologous to the Golgi outposts observed in neurons^59^,^60^,^61^, responsible for the trafficking and processing of membrane proteins to locations distant from the cell body. Our findings raise two key questions regarding the basal process. First, are newly synthesized plasma membrane constituents delivered to the basal process via the canonical ER–Golgi–plasma membrane route, even though this would obligatorily imply either vesicular transport or lateral diffusion in the plane of the plasma membrane from the apical process over hundreds of microns to as far as the pial endfoot of the basal process? Alternatively, and in light of the presence of ER and ERES but the absence of Golgi apparatus in the basal process, do aRGs exploit the option of the unconventional route of biosynthetic vesicular traffic to the plasma membrane that bypasses the Golgi apparatus? Neurons do provide a classical example that the function of the plasma membrane (at the presynaptic terminal) can be perfectly maintained when the Golgi apparatus is not only hundreds of microns, but as much as meters away^62^,^63^. However, neurons are post-mitotic cells with a lifespan of decades, whereas the duration of a cell cycle of aRGs is a day or less^64^. This difference may pose rather different requirements for the machinery mediating the delivery of newly synthesized plasma membrane constituents.

Our observation that the plasma membrane constituents of the aRG basal process contain ER-derived but largely lack Golgi-derived glycan moieties indeed suggests that these constituents are delivered from the ER, which is abundantly present in the basal process, via the unconventional route of biosynthetic membrane traffic directly to the plasma membrane, bypassing the Golgi apparatus located in the apical process^46^,^49^,^65^. In this context, it is interesting to note that integrins, which have been implicated in transducing signals that promote neural stem and progenitor proliferation and self-renewal, and which have been found to be located on the basal process, have been shown to be delivered to the plasma membrane via the unconventional biosynthetic membrane traffic pathway^66^,^67^. Considering the spatial distances inherent in the aRG architecture, this unconventional route provides an economical way of ensuring basal process growth and maintenance in this rapidly replicating cell.

**Figure 12:**
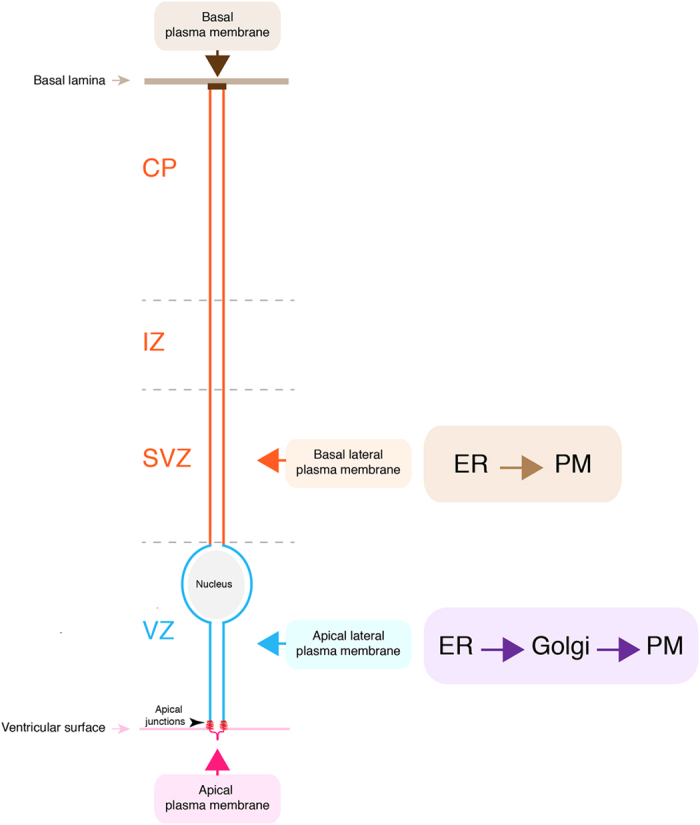
Cartoon illustrating the distinct aRG plasma membrane domains and the canonical vs. unconventional biosynthetic membrane traffic routes to these domains. Membrane constituents of the apical plasma membrane domain (magenta) and the apical lateral plasma membrane domain (blue) are largely delivered via the canonical traffic route (purple), transiting through the Golgi apparatus; PM, plasma membrane. In contrast, membrane constituents of the basal lateral plasma membrane domain (orange) and the basal plasma membrane domain (dark brown) are proposed to be largely delivered via the unconventional traffic route (light brown), bypassing the Golgi apparatus. IZ, intermediate zone. It should be noted that the presence of ConA-stained membrane constituents in the apical and the apical lateral plasma membrane domains (see Fig. 11E and Supplementary Figure S7A) may reflect either traffic via a Golgi-independent route or lack of processing of high-mannose N-linked glycans during their passage through the Golgi apparatus.

Furthermore, our data imply that the lateral plasma membrane of aRGs is not created equal along the apical-basal axis of the developing cortical wall, but rather is highly sub-compartmentalized (Fig. 12). This sub-compartmentalization is intriguing, as it mirrors the histological compartmentalization of the developing neocortex, i.e. its subdivision into the VZ, the primary germinal layer, on the one hand and the SVZ, intermediate zone and CP as secondary cell layers on the other hand. In fact, our data suggest that Golgi-derived versus directly ER-derived traffic to the plasma membrane differentially contribute to the biogenesis and maintenance of the apical versus basal process. Thus, the Golgi apparatus provides membrane mainly to the apical process, including both the apical plasma membrane proper and the apical lateral plasma membrane, but not to the basal process with its basal lateral plasma membrane and the basal plasma membrane at the pial end-foot (Fig. 12).

Although speculative at present, it may be worthwhile to consider the presence of ER-derived but the lack of Golgi-derived glycan moieties on the surface of the aRG basal process in the context of neuronal migration^68^,^69^. Newborn cortical neurons, most of which are generated in the SVZ, use the basal process as a guiding structure to migrate through the intermediate zone to the CP^70^. Perhaps there are specific features of molecular interaction between the directly ER-derived glycan moieties on the aRG basal process and the Golgi-derived glycan moieties on the neuronal surface that have a role in neuronal migration to the CP.

## Conclusion

aRG and the basal radial glia derived therefrom are polarized epithelial stem cells with pivotal roles in cortical development. The present study has elucidated a key aspect of aRG cell biology that is relevant for their bipolar epithelial nature – the organization, dynamics and functional contribution of the Golgi apparatus in these cells. Moreover, we have demonstrated the unique features of the basal process, a hallmark of both aRG and basal radial glia. It is a probable forecast that further dissection of polarized membrane traffic in these neural stem cells will provide important contributions towards understanding the basis of their proliferative and self-renewal capacity that is so crucial for neocortex expansion in development and evolution.

## Materials and Methods

### Mouse lines

Embryonic day (E) 13.5-14.5 C57BL/6 mouse embryos were used as representative of mid-neurogenesis time window. BAC-transgenic *Tbr2(Eomes)*-GFP^10^, transgenic *GFAP*-GFP^18^ and *Tubb3*-GFP^71^ were maintained on a C57 B6/J background, and hemizygous E13.5-E14.5 embryos, validated under a fluorescence dissection microscope, were used. Mouse lines were maintained in pathogen-free conditions in the animal facility of the Max Planck Institute for Molecular Cell Biology and Genetics, Dresden, Germany. All experiments were performed in accordance with German animal welfare legislation. Protocols were first internally approved by the Institutional Animal Welfare Officer, and formal approval and the necessary licenses were subsequently obtained from the regional (that is, Dresden, Germany) Ethical Commission for Animal Experimentation (Tierversuchskommission according to §15 of the German Animal Welfare Act, Landesdirektion Sachsen).

### EdU labeling *in vivo*

*In vivo* EdU labeling was carried out by intraperitoneal injection of 1 mg EdU (Sigma) in 100 μl PBS into pregnant females 13.5 days post-coitum^64^. Mice were sacrificed 30 min after injection, and embryos were collected in ice-cold PBS in order to stop the EdU incorporation. Embryos were fixed with 4% paraformaldehyde (PFA), processed for vibratome sectioning (see below) and EdU was detected using the Click-It kit (Molecular Probes) according to the manufacturer’s instructions.

### DiI labeling

For DiI labeling of aRG^51^, a DiI working solution was prepared by dissolving DiI crystals (Molecular Probes, D-3911) in 1 ml DMSO (Sigma) to a final concentration of 0.2 mg/ml. The resulting solution was mixed by vortexing, and then centrifuged at 16,000 × g, for 30 min at room temperature. The supernatant was transferred to a new tube and stored in the dark at room temperature for a maximum of 1 month.

In these experiments, C57 B6/J wt E14.5 mouse embryos were used. Embryonic brains were dissected, and cerebral hemispheres separated from each other, in ice-cold Tyrode solution (TS). After dissection, hemispheres were kept on ice until transferred to a petri dish filled with 10–15 ml of pre-warmed (37 °C) TS, and DiI (2–5 μl) was selectively and sparsely applied to the ventricular surface of the cortical tissue by means of a mouth-controlled pipette-tip. Next, the hemispheres were transferred to a new TS-filled petri dish, and unbound DiI crystals washed away by gentle rotation of the dish. During the entire procedure, specificity and magnitude of labeling were visualized by epifluorescent illumination under a stereomicroscope (SZX16 Olympus). After DiI application, each mouse cerebral hemisphere was transferred to a 4% PFA-filled tube and placed on a shaker at room temperature for 2–3 h.

### Assessment of cell cycle phases for aRGs

Throughout these experiments, E13.5 *GFAP*::GFP mice were used. The different cell cycle phases were discriminated as follow.

#### S- and G2-phase

We made use of HERO-Culture system^26^,^35^, coupled with an EdU pulse-chase paradigm to identify cells in S- and G2-phase, respectively. A 30 min pulse followed by immediate fixation allows identifying S-phase cells^35^. Cells in G2 could be identified by a 30 min EdU pulse followed by a chase in the absence of EdU and fixation^35^.

Briefly, embryonic brains were dissected in TS at room temperature, and cerebral hemispheres separated from each other. Meninges were removed surgically and the hemispheres were transferred to 20 ml glass flasks (1 brain per flask) containing 1.2 ml of slice culture medium (SCM) (Attardo *et al.*, 2006), consisting of Neurobasal medium (Gibco) supplemented with 10% rat serum (Charles River Japan), 2 mM L-glutamine (Gibco), Penstrep (Gibco), N2 supplement (17502-048, Invitrogen), B27 supplement (17504-044, Invitrogen) and 10 mM Hepes. Hemispheres were allowed to equilibrate for 30–40 min in a whole-embryo culture incubator (RKI Ikemoto) at 37 °C in an atmosphere of 40% O2, 5% CO2, 55% N2, with continuous rotation at 20 rpm. After equilibration, EdU was added from a stock solution to the SCM at a final concentration of 1 mg/ml. After 30 min (pulse) brains were thoroughly washed in warm Tyrode solution, and then either immediately fixed in 4%PFA (T = 0) or transferred to flasks with fresh, prewarmed, and oxygenated SCM and fixed after 1 h, 1 h 30 min, or 2 h (chase). Brains were further processed for vibratome sectioning and immunofluorescence.

#### G1-phase

Cells in late G1 were identified by their positivity to CyclinD1, while cells in early G1 were identified as pairs of daughter cells (with similar nuclear features) close to the apical surface.

#### M-phase

Cells in M-phase were identified by chromosome condensation and their localization at the apical surface, and were scored according to their progression through different mitotic phases as judged by DAPI staining.

### Histology, immunofluorescence staining and image acquisition

Samples (either brains or brain hemispheres) were fixed with 4% PFA in 120 mM phosphate buffer pH 7.4 for 30 min at room temperature followed by overnight incubation at 4 °C^64^,^72^. The fixed samples were embedded in 3% agarose in PBS and subjected to vibratome sectioning at 50–80 μm. Vibratome sections were collected and stored in PBS at 4 °C until further processing.

### Immunofluorescence staining

Immunofluorescence staining of vibratome sections was performed by permeabilizing with 0.3% Triton X-100 in PBS for 30 minutes, quenching with 2 mM glycine in PBS for 30 minutes, followed by blocking, primary and secondary antibody incubations, and washing in a solution of 0.2% gelatin, 300 mM NaCl and 0.3% Triton X-100 in PBS. The primary antibody was incubated for 2 days at 4 °C. For giantin and Pax6 staining, immunofluorescence was performed after antigen retrieval by incubating at 70 °C for 30 min in 0.01 M Na-Citrate.

The following primary antibodies were used: α-tubulin clone B-5-1-2 (mouse mAb, T5168, 1:1000, Sigma), Cyclin D1 (mouse monoclonal, sc-450, 1:200, Santa Cruz), γ-tubulin (mouse mAb, T6557, 1:400; rabbit polyclonal, T51892, 1:400; both from Sigma), TuJ1 (mouse mAb, MMS-435P, 1:200, Covance), pan-cadherin (mouse mAb, C1821, 1:500, Sigma), GFP (goat polyclonal, 1:1000, MPI-CBG), GRASP-65 (rabbit polyclonal, ab30315, 1:2000, Abcam), giantin (mouse mAb, ALX-804-600, 1:100, Alexis), KDEL (Rabbit polyclonal antibody recognizing the KDEL sequence, 1:100, Abcam), Pax6 (rabbit polyclonal, PRB-278P, 1:200, Covance), Sec 16 (rabbit polyclonal anti-KIAA0310, 1:100, Bethyl), Sec61A (rabbit polyclonal, Ab15575, 1:200, Abcam), Tbr2 (rabbit polyclonal, AB2283, 1:200, Millipore). Primary antibodies were followed by 1h incubation at room temperature with the appropriate A488-, A594-, A555- or A647-labeled secondary goat or donkey antibodies (Alexa series, 1:1000, Invitrogen). Floating sections were stained with 4,6-diamidino-2-phenylindole (DAPI, Roche) and mounted in Mowiol 4-88. Samples were analyzed by confocal microscopy (Zeiss Axiovert 200M LSM 510, Zeiss, Jena).

### Plasma membrane lectin staining

To detect plasma membrane glycoconjugates in fixed brain tissue, 70–80 μm thick vibratome sections were incubated for 20 h at room temperature with fluorescently-conjugated ConA-Alexa-488 and/or WGA-Alexa-488 (Molecular Probes) in PBS containing 0.2% gelatin and 300 mM NaCl. DAPI was added as a counterstain.

### Electron microscopy

For conventional transmission electron microscopy^30^, fixed mouse embryos at E13.5 were embedded in 4% low-melting agarose (ScienceServices) in PBS (containing 0.5 mM MgCl_2_ and 0.9 mM CaCl_2_), followed by the preparation of transverse 200-μm vibratome sections (VT1200S, Leica). Mouse embryonic sections or pieces of fixed adult mouse duodenum were post-fixed in 1% osmium tetroxide containing 1.5% potassium ferrocyanide for 30 min at room temperature, stained with 0.5% tannic acid for 10 min and with 1% osmium tetroxide for 30 min and subsequently contrasted with 1% uranyl acetate, dehydrated and flat-embedded in Epon replacement (Carl Roth).

Ultrathin (70 nm) plastic sections of dorsal telencephalon or duodenum were cut on a (Leica, UCT) microtome and post-stained with uranyl acetate and lead citrate according to standard protocols. Images were taken on a Morgagni EM at 80 kV (FEI) with a Morada camera (Olympus) and ITEM software (Olympus).

For serial block face scanning electron microscopy (SBF-SEM) E12.5 mouse embryos were fixed and vibratome-sectioned as described in Paridaen *et al.*^31^. The 200 μm-sections were post-fixed in 2% osmium tetroxide containing 1.5% potassium ferrocyanide for 30 min at room temperature, stained with 1% thiocarbohydrazide for 15 min, fixed with 1% osmium tetroxide for 30 min, stained with 1% thiocarbohydrazide for 20 min and incubated again with 1% osmium tetroxide for 30 min. Samples were subsequently contrasted with 0.5% uranyl acetate in 25% methanol overnight at 4 °C and with Walton’s lead acetate for 30 min at 60 °C. After ethanol dehydration, the samples were infiltrated and flat-embedded in a hard Epon-replacement resin. A small portion of the dorsal telencephalon was mounted on a pin, pre-trimmed in a microtome and placed in a scanning electron microscope (FEI, Magellan 400) equipped with an internal microtome (Gatan, 3View2XP). Serial sectioning was performed at 50 nm steps. Serial backscattered electron images (1.9 kV, 100 pA, immersion mode) of the block face were recorded after each 50 nm-section at 9 nm/pixel resolution^24^ (24 images per section). The 24 images per section were compiled into a montage, and the montages of the serial sections were aligned and analysed using the TrakEM2 plugin in Fiji software^73^. Immuno-EM was performed as described in Paridaen *et al.*^31^.

### In utero electroporation

In utero electroporation was carried out as previously described^31^. Briefly, mice carrying E13.5 embyos were anesthetized using isoflurane. After the administration of painkiller, the abdominal cavity was open and the uterus was exposed. A solution containing the plasmids of interest and FastGreen as a tracer was injected intraventricularly using a glass pipette (pCS2-GAP43-GFP and pCS2-GalNacCherry: 0.4 μg/μl; pCAGGs-Cherry, GM130-GFP and pCS2-GalNacGFP 0.3 μg/μl; pCS2-GalNacCherry and pCS2-GalNacGFP was obtained from Paolo Ronchi and Rainer Pepperkok, EMBL). The electroporation was achieved using a plate electrode and applying 5–6 40 V pulses, 50 ms in length. After electroporation, the uterus was relocated in the peritoneal cavity, and the abdomen was sutured. The embryos were allowed to develop for 24 h. The pregnant female was then sacrificed by cervical dislocation and the embryos were collected in warm Tyrode solution. The dissected brains were assessed for GFP positivity using a dissectoscope equipped with fluorescent filters, and the positive hemispheres were either fixed immediately in 4% PFA for immunofluorescence analysis (see also above), or further processed for slice culture and live imaging.

### Slice culture

After surgical removal of meninges, hemispheres were immersed in 3% low melting agarose in PBS (Agarose Type XI, Sigma) at 37 °C. After letting agarose solidify upon cooling to room temperature, 180 μm-thick slices were cut using a vibratome (Leica VT1200S, Leica), with the cutting plane being oriented perpendicular to the rostro-caudal axis of the hemisphere^26^. Slices were transferred to a silicon-coated 10 cm dish containing 37 °C-warm SCM. Positive slices were selected using a dissectoscope equipped with fluorescent filters. The ventral-most part of the ganglionic eminence was removed using a micro knife, and the resulting slices were embedded in collagen. Collagen-immersed slices were transferred with <200 μl collagen solution into the 14 mm well of a 35 mm Glass-Bottom-Microwell-Dish (MatTek Corporation, Ashland, USA). The dish was placed for 5 min at 37 °C, followed by ≥40 min in the slice-culture incubator to allow the collagen to solidify.

### Live imaging

Organotypic slice culture imaging was performed with a Zeiss LSM 710 NLO laser-scanning microscope, equipped with a 40 × C-Apochromat 1.2 N.A. W objective (Carl Zeiss), as described^26^. After collagen solidification, the dishes with the sections to be imaged were kept on the microscope stage at 37 °C, in 1.5 ml of SCM. During live imaging, Slice culture was maintained with an air flow of 40% O_2_, 5% CO_2_, 55% N_2_. Stacks of typically 1024 × 1024 pixels × 20 optical sections (xyzt sampling: 0.35 × 0.35 × 2.5 μm × 20 min) were acquired for ∼24 h. Potential phototoxicity effects were stringently controlled as described^74^.

### Zebrafish husbandry

WT AB and WT TL were used. Zebrafish were maintained and bred at 26.5 °C. Embryos were raised at 28 °C and treated with 0.003% phenylthiourea (Sigma) from ~8 hpf to prevent pigmentation. All animal work was performed in accordance with EU directive 2011/63/EU as well as the German Animal Welfare Act.

### Zebrafish constructs

pCS2 + GFP/RFP-PCNA, pCS2 + GFP-Centrin, pCS2 + H2B-RFP, pCS2 + mKate2-Ras, pCS2 + EB3-mKate2 and hsp70-mKate2 were published previously^32^,^75^,^76^,^77^. pCS2 + CFP-tagged beta-galactosyltransferase (GalTase-CFP) was a kind gift of William Harris, University of Cambridge. Tol2-h2ax-man2A-eGFP^78^ (Mannosidase-GFP) was kindly provided by Brian A. Link, Medical College of Wisconsin. pCS2 + GFP-GM130 was a kind gift of Virginie Lecaudey, University of Freiburg.

Tol2 Gateway kit^79^ was used to create Tol2-hsp70-stathmin1-mKate2, Tol2-hsp70-mKate2-dynactin(1-811) and Tol2-hsp70-mKate2-DNKif5a. The mKate2 middle entry clone was a gift from Andrew Oates lab. The coding sequence of human stathmin 1 (oncoprotein 18) was a gift from Konrad Bussow^80^ (Addgene plasmid # 31326). The stathmin 1 middle entry clone was created by PCR using Phusion polymerase (NEB) with primers:

5′-ggggacaagtttgtacaaaaaagcaggctggATGGCTTCTTCTGATATCCA-3′

5′-ggggaccactttgtacaagaaagctgggtcGTCAGCTTCAGTCTCGTCAG-3′

The coding sequence of human dynactin subunit 1 (p150 Glued) was a gift from Claude Prigent^81^. The dynactin1-811 3′ entry clone was created with primers:

5′- ggggacaagtttgtacaaaaaagcaggctggATGGTGAGCGAGCTGATTAAGG-3′

5′- ggggaccactttgtacaagaaagctgggtcTTAAGGAGCATCTGTCCCT-3′

The N-terminally truncated Kif5aa (kinesin-1, NM_001199776) was amplified from zebrafish cDNA to create DNKif5a 3′entry clone (missing first 415 amino acids, 416-1033 truncation) with primers:

5′- ggggacagctttcttgtacaaagtggctTCCTCTATCGTGGTGCGC-3′

5′- ggggacaactttgtataataaagttgcTTAACTGGCTGCAGTCTCC-3′

### Zebrafish RNA injection

RNA was synthesized using the Ambion SP6 mMessage mMachine kit. RNA (50–125 ng/μl) was injected in volume of 0.3–0.6 nl into one to two cells of 16–64 cell stage zebrafish embryos.

### Zebrafish DNA injection and heat shock

Plasmid DNA was diluted in water supplemented with 0.05% Phenol red and was injected into the cytoplasm of the one-cell stage zebrafish embryos. The volume injected ranged from 0.5 to 1 nl. The total DNA concentration was 30 ng/μl of which 20 ng was one of the heat shock constructs and 10 ng was the Mannosidase-GFP. The embryos were heat shocked at 24 hpf for 30 min in a water bath at 37 °C and imaged 4 hours later live using a confocal microscope.

### Zebrafish *in vivo* time lapse

For *in vivo* time-lapse imaging, zebrafish embryos were anaesthetized using 0.04% MS-222 (Sigma) and mounted in 1% low melt agarose in E3 medium on MatTek glass bottom dishes. An Andor spinning disk system with an Olympus UPlanSApo 60x water immersion objective (NA = 1.2) was used. A heating chamber at 30 °C was used. Z stacks of 28–30 μm were collected and optical sections were 1 μm. Images were acquired every 5 min for 10 h–18 h. Time-lapses were started at ~28 hpf.

### Image analysis and quantification

#### Mouse

Image analysis was performed using Fiji software.

#### Single cell analysis

For all cells analyzed, the cell’s contours were traced in every single optical section using the GFP (immune)fluorescence. Cell contours were then used to identify the DAPI-stained nucleus, the γ-tubulin–stained centrosome(s), the Golgi apparatus (as revealed by the GRASP-65 immunoreactivity), the absence or presence of CyclinD1 immunoreactivity and the EdU positivity. For the nucleus and the centrosome, he distance from the apical plasma membrane was calculated. For the Golgi apparatus, we measured the distance between the apical-most and the basal-most Golgi unit from the apical surface.

#### Profile plot

For the graph shown in Fig. 3B, the GRASP-65 and EdU staining across the cortical wall were quantified using the profile plot tool of Fiji on a 20-μm-wide window. The window height was set to match the height of the cortical wall.

#### Line plot

For the quantifications shown in Fig. 11, the line plot tool of Fiji was used. The line was placed blind using the DiI channel, and its position was recorded using the ROI manager. The recorded positions were then used to quantify the lectin signal.

#### Mouse duodenum

For the analysis of the position of Golgi stacks relative to the nucleus and centrosome, electron micrographs of sections through 38 progenitor cells in intestinal crypts (each displaying a Golgi stack, nucleus, centriole and apical plasma membrane in a single plane) were acquired. The distance between these organelles was determined by the measure function in the ITEM software (Olympus).

#### Zebrafish

Tracking was performed manually by measuring the distance between the apical surface and the center of the nucleus or the apical-most and the basal-most Golgi. Tracking was performed during different cell cycle stages as assessed by the appearance of PCNA marker^32^. Additionally, ManualTracking Plugin in Fiji software was used to visualize nuclear and Golgi dynamics around the entry into G2 phase and initiation of rapid apically directed migration. Matlab software (MathWorks, Inc) was used to prepare graphs.

## Additional Information

**How to cite this article**: Taverna, E. *et al.* Non-canonical features of the Golgi apparatus in bipolar epithelial neural stem cells. *Sci. Rep.* **6**, 21206; doi: 10.1038/srep21206 (2016).

## Supplementary Material

Supplementary Information
