# Supplementary material for: Non-canonical features of the Golgi apparatus in bipolar epithelial neural stem cells
Source: Sci Rep. 2016 Feb 16;6:21206. doi: 10.1038/srep21206 (PMC4754753; doi:10.1038/srep21206)
Supplement: Supplementary Information [file srep21206-s1.pdf]

Non-canonical features of the Golgi apparatus in bipolar epithelial neural stem cells

Elena Taverna*, Felipe Mora-Bermúdez, Paulina J. Strzyz, Marta Florio,

Jaroslav Icha, Christiane Haffner, Caren Norden,

Michaela Wilsch-Bräuninger and Wieland B. Huttner*

Max-Planck Inst. of Mol. Cell Biol. and Genetics

Pfotenhauerstr. 108, 01307 Dresden, Germany

*Corresponding authors

taverna@mpi-cbg.de

huttner@mpi-cbg.de

Phone: +49-351-210-1500

Fax: +49-351-210-1600

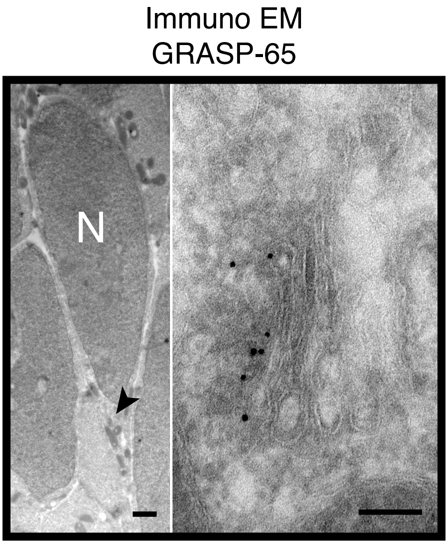

Supplementary Figure S1. Immuno-gold electron microscopy of the aRG Golgi apparatus. Left micrograph, Golgi apparatus (arrowhead) located apical to an aRG nucleus (N). Right micrograph, high magnification of the Golgi stack indicated in the left micrograph, labeled by anti-GRASP-65 antibody and 10 nm immuno-gold. Scale bars, 1 μm (left) and 100 nm (right).

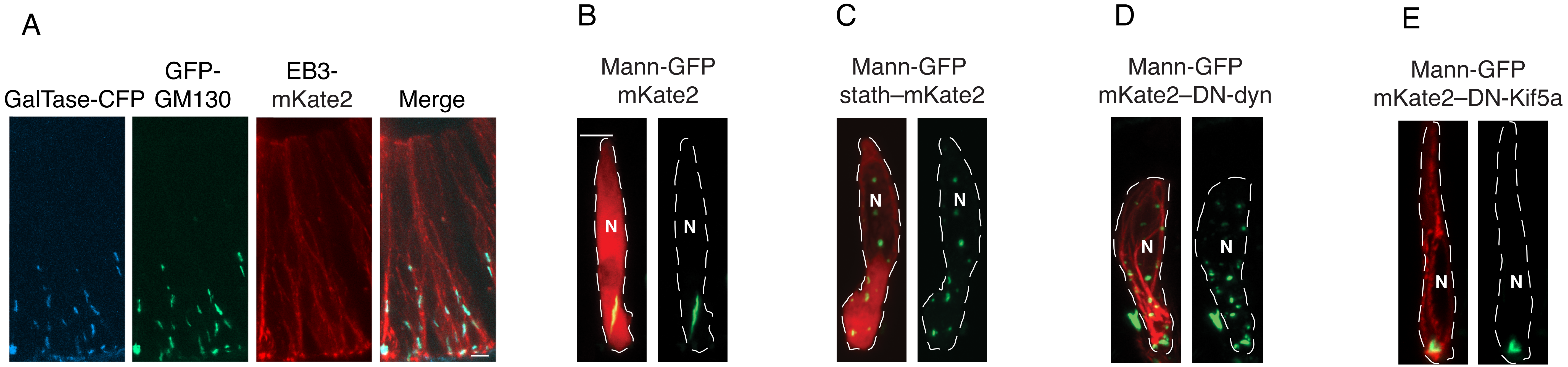

Supplementary Figure S2. Role of microtubules and microtubule-based motors in the apical localization of the Golgi apparatus in zebrafish retinal neuroepithelial cells.

A. Pattern of the Golgi apparatus in relation to microtubules in zebrafish retinal neuroepithelial cells. Embryos were injected at the 16-64–cell stage with the indicated in vitro transcribed mRNAs and analyzed at 28 hpf. Confocal image (stack of 6 1-μm optical sections) showing the localization of the Golgi apparatus, as revealed by expression of GalTase–CFP (cyan) and GFP-GM130 (green), relative to microtubules, as revealed by expression of EB3-mKate2 (red). Scale bar, 5 μm.

B-E. Zebrafish embryos at the one-cell stage were co-injected with DNA coding for mannosidase-GFP to reveal the Golgi apparatus and with the indicated heat-shock inducible constructs, followed by heat shock at 24 hpf and analysis of retinal neuroepithelial cells (dashed lines) 4 hours later. Apical surface is down. Scale bar, 5 μm.

B. Golgi apparatus localization (green) in a control cell, the shape of which is revealed by expression of soluble mKate2. Note the Golgi localization apical to the nucleus (N). Images are stacks of 6 1-μm optical sections.

C. Dispersion of the Golgi apparatus upon destabilization of microtubules by expression of stathmin (stathmin-mKate2). Note some Golgi units basal to the nucleus (N). Images are stacks of 6 1-μm optical sections.

D: Dispersion of the Golgi apparatus upon inhibition of dynein activity by expression of dominant-negative dynactin (mKate2–DN-dyn). Note some Golgi units basal to the nucleus (N). Images are stacks of 6 1-μm optical sections.

E. Compaction of the Golgi apparatus apical to the nucleus (N) upon inhibition of the kinesin-1 motor protein by expression of dominant-negative Kif5a (mKate2–DN-Kif5a). Images are stacks of 6 1-μm optical sections.

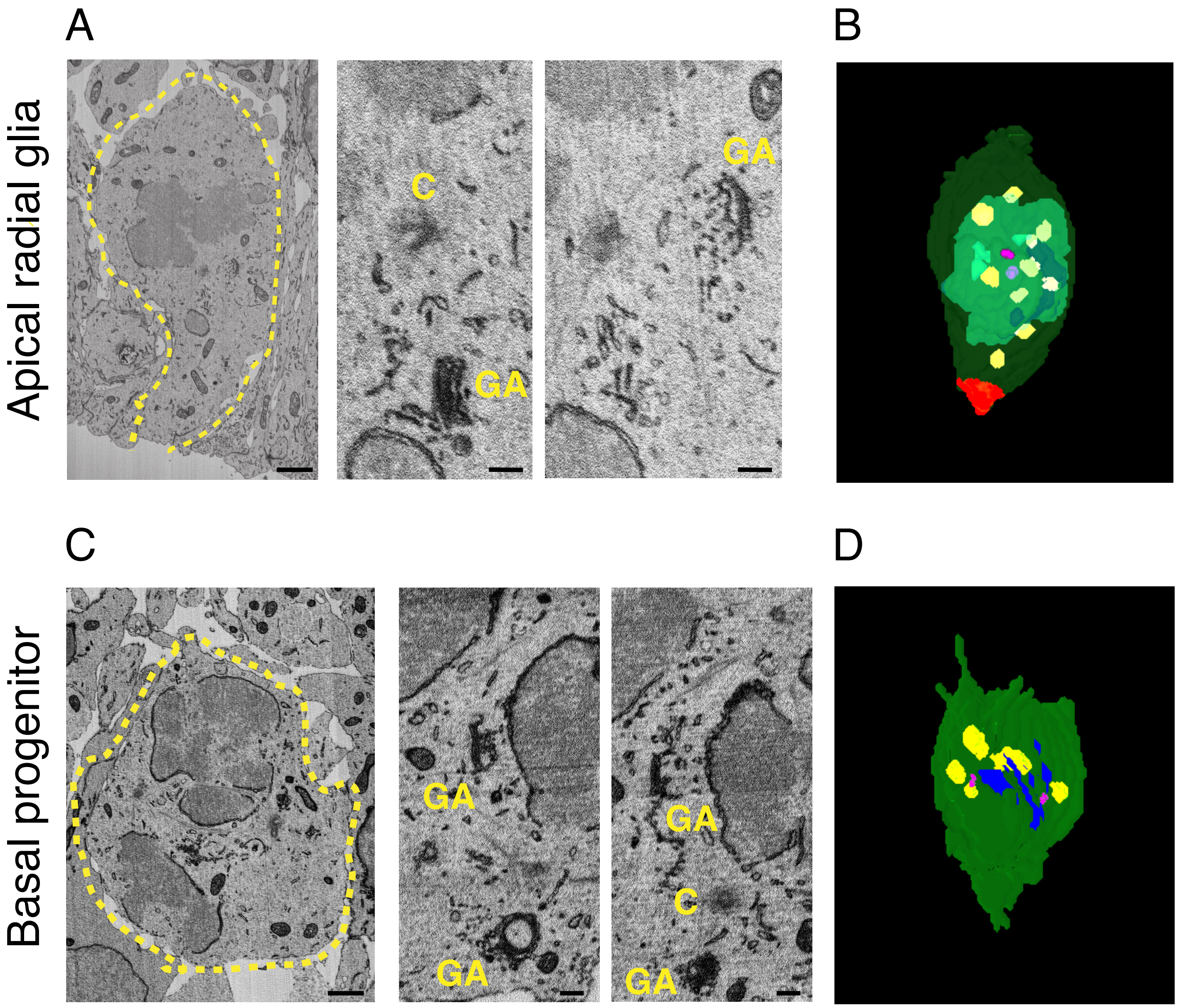

Supplementary Figure S3. SBF-SEM of an aRG and a bIP in metaphase.
Early metaphase aRG (**A, B**) and bIP (**C**, **D**) in E12.5 wt mouse dorsolateral telencephalon. (**A**, **C**) Left images, low magnification; middle and right images, adjacent sections showing the Golgi apparatus (GA) at higher magnification. Note the proximity of Golgi units to the centrosome (C). (**B, D**) SBF-SEM 3D reconstruction of the Golgi apparatus in the entire cell body of the aRG (**B**) and bIP (**D**) shown in (**A**) and (**C**), respectively. Green, cytoplasm; blue, chromatin; magenta, centrosomes; yellow, Golgi units; orange, apical plasma membrane. Scale bars, low magnification 2 μm, high magnification 500 nm.

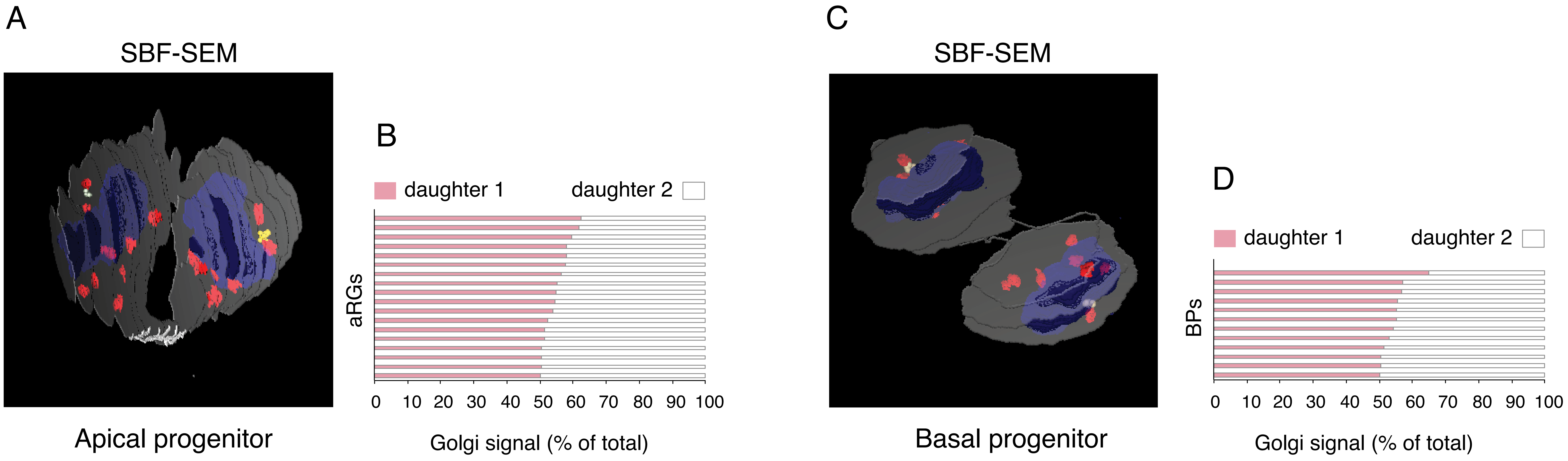

Supplementary Figure S4. Golgi apparatus partitioning in mitotic APs and BPs.
A, C. SBF-SEM 3D reconstruction of the Golgi apparatus partitioning in a telophase aRG (**A**) and bIP (**B**) in E12.5 wt mouse dorsolateral telencephalon. Red, Golgi apparatus; blue, nucleus; yellow, centrosomes; white, apical plasma membrane.
B, D. Dorsolateral telencephalon of E13.5 GFAP::GFP (**B**) or Eomes::GFP (**D**) transgenic mouse embryos was (immuno)stained for GFP (to identify individual aRGs and BPs, respectively), GRASP-65 (to reveal the Golgi units), cadherin (to reveal the cell contours) and DAPI (to assess the mitotic phase). Each of the indicated aRGs and BPs in either anaphase or early telophase was analyzed to integrate the Golgi immunofluorescence signal in the prospective daughter cell pairs (total), and the distribution of the Golgi signal between the two daughter cells is expressed as percent of total.

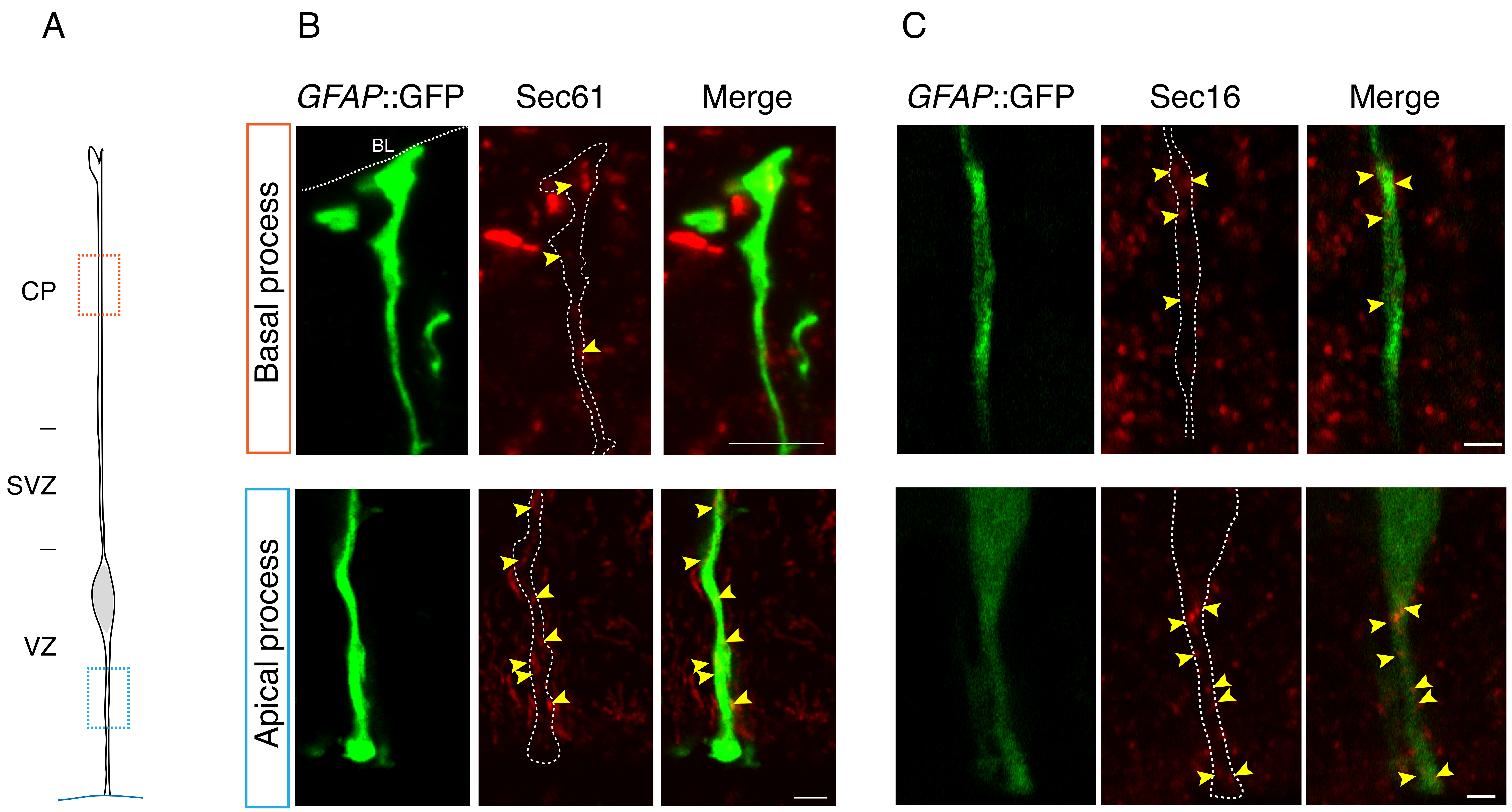

Supplementary Figure S5. Both apical and basal process of aRGs contain rough ER.
A. Cartoon of an aRG illustrating the region of the apical and basal process subjected to immunofluorescence analysis in (**B** and **C**). Blue box, apical process in the VZ apical to the nucleus; orange box, basal process in the CP.
B, C. High magnification of portions of the basal process (top row) and the apical process below the nucleus (bottom row) of single aRGs in dorsolateral telencephalon of E14.5 GFAP::GFP transgenic mouse embryos, identified by GFP immunofluorescence (green), showing the presence of rough ER (arrowheads) as revealed by immunofluorescence for Sec61 (**B**, red) and Sec16 (**C**, red). Dashed lines indicate the apical or basal process of the aRG analyzed. Dotted line in (**B**, left panel), basal lamina (BL). Images are single 0.6-μm optical sections. Scale bars, 2 μm.

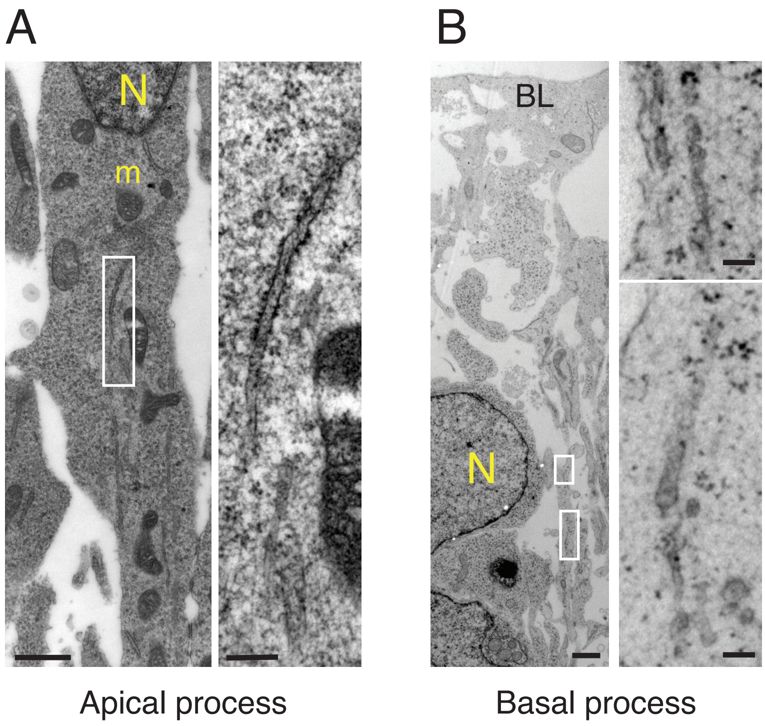

Supplementary Figure S6. Transmission electron microscopic analysis corroborates the presence of rough ER in the apical and basal process.

A. Left micrograph, apical process near the ventricular surface (down) in embryonic wt mouse dorsolateral telencephalon. Note the aRG nucleus (N) at the top; m, mitochondrion. The white box indicates the region presented at higher magnification in the right micrograph, showing the presence of rough ER. Scale bars, low magnification 1 μm, high magnification 100 nm.

B. Left micrograph, basal process (white boxes) in the CP in embryonic wt mouse dorsolateral telencephalon. Note the basal lamina (BL) at the top. N, neuronal nucleus. The regions in the white boxes are presented at higher magnification in the right micrographs, showing the presence of rough ER. Scale bars, low magnification 1 μm, high magnification 100 nm.

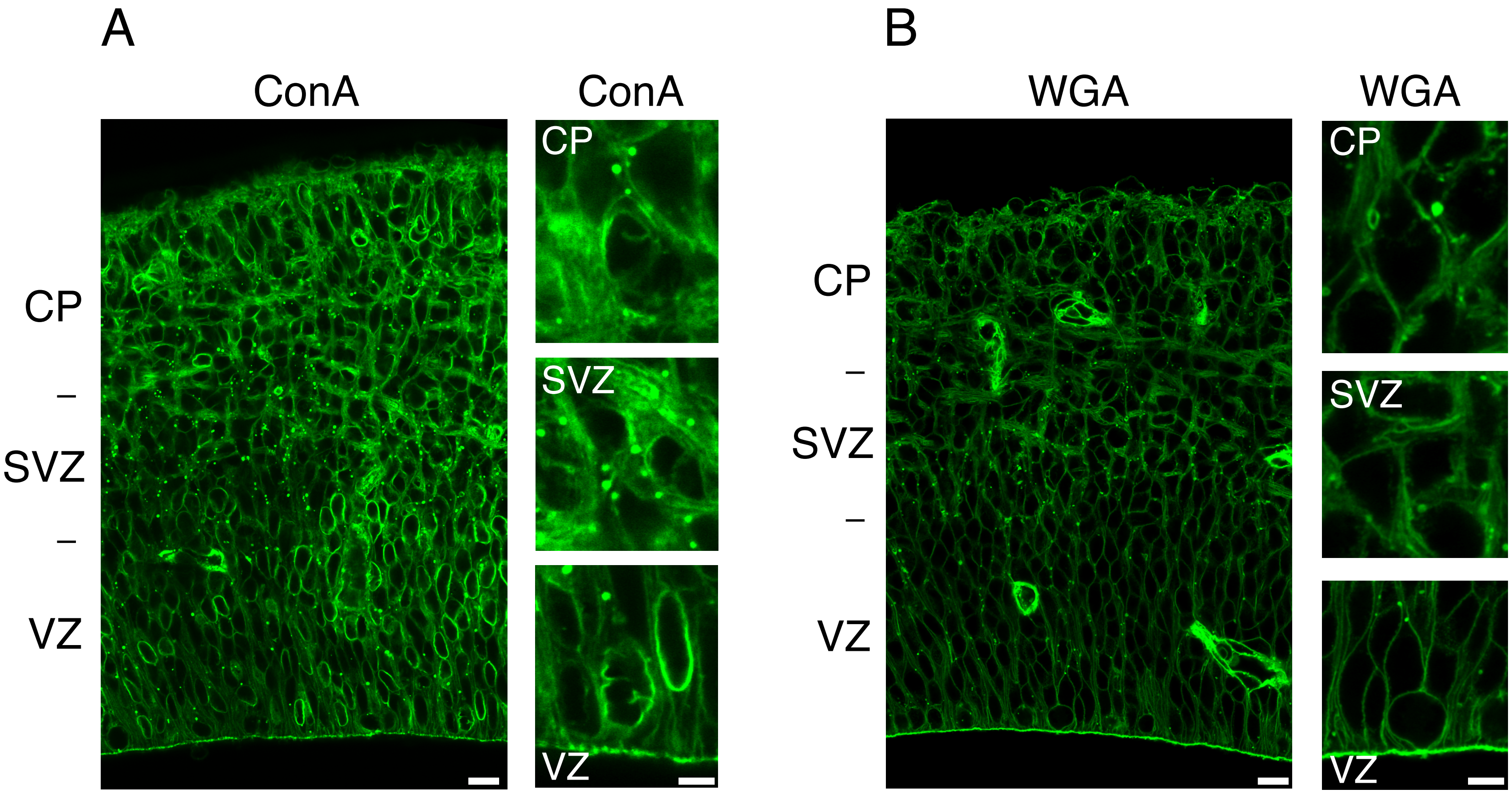

Supplementary Figure S7. Lectin staining of the E14.5 wt mouse neocortex.
ConA-488 (A) and WGA-A488 (B) cell surface staining. Right panels show a higher magnification of VZ, SVZ and CP. Note that the lectin-stained spots, which are particularly abundant upon ConA-488 staining, presumably reflect extracellular lipoprotein particles. Images are 0.6-µm single optical sections. Scale bars: low magnification, 10 µm; high magnification, 5 µm.

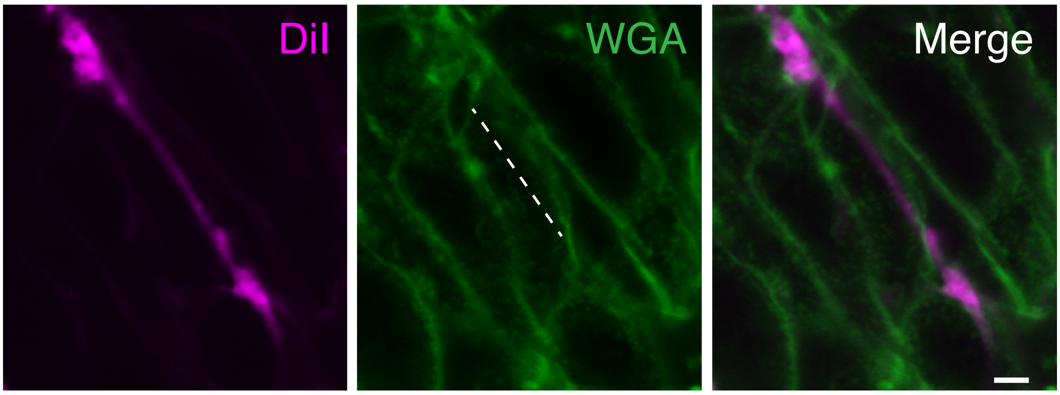

Supplementary Figure S8. The plasma membrane of the aRG basal process lacks Golgi-modified glycans.
E16.5 wt mouse cerebral hemisperes were labeled with DiI (magenta) applied to the ventricular surface, fixed and cell surface-stained with WGA-A488 (green). Images show a DiI-labeled basal process, the position of which is indicated by a dashed white line in the WGA image, and are single 0.6-μm optical sections. Scale bar: 2 μm.
